# Mycobacteria Modify Their Cell Size Control under Sub-Optimal Carbon Sources

**DOI:** 10.3389/fcell.2017.00064

**Published:** 2017-07-12

**Authors:** Miles Priestman, Philipp Thomas, Brian D. Robertson, Vahid Shahrezaei

**Affiliations:** ^1^Department of Medicine, MRC Centre for Molecular Bacteriology and Infection, Imperial College London London, United Kingdom; ^2^Department of Mathematics, Imperial College London London, United Kingdom

**Keywords:** cell size control, mycobacteria, asymmetric cell division, adder, sizer

## Abstract

The decision to divide is the most important one that any cell must make. Recent single cell studies suggest that most bacteria follow an “adder” model of cell size control, incorporating a fixed amount of cell wall material before dividing. Mycobacteria, including the causative agent of tuberculosis *Mycobacterium tuberculosis*, are known to divide asymmetrically resulting in heterogeneity in growth rate, doubling time, and other growth characteristics in daughter cells. The interplay between asymmetric cell division and adder size control has not been extensively investigated. Moreover, the impact of changes in the environment on growth rate and cell size control have not been addressed for mycobacteria. Here, we utilize time-lapse microscopy coupled with microfluidics to track live *Mycobacterium smegmatis* cells as they grow and divide over multiple generations, under a variety of growth conditions. We demonstrate that, under optimal conditions, *M. smegmatis* cells robustly follow the adder principle, with constant added length per generation independent of birth size, growth rate, and inherited pole age. However, the nature of the carbon source induces deviations from the adder model in a manner that is dependent on pole age. Understanding how mycobacteria maintain cell size homoeostasis may provide crucial targets for the development of drugs for the treatment of tuberculosis, which remains a leading cause of global mortality.

## 1. Introduction

Bacteria have long been known to maintain their average size within steady state populations, but we do not yet have a full mechanistic understanding of how this is achieved (Chien et al., 2012). The average bacterial size is a function of growth condition and it has been suggested that it scales exponentially with the population growth rate (Schaechter et al., 1958). Cell size homoeostasis and its interplay with gene expression and growth is of critical importance for bacterial survival, since the requirement for additional space to incorporate nascent DNA and to subsequently permit cell division must be balanced with the necessity to maintain the concentration of critical proteins, such as those involved in metabolism or protein synthesis (Shahrezaei and Marguerat, 2015). At the single-cell level, size homoeostasis is implemented through the strategy cells employ to decide when to divide, which sets their average final size as well as their average birth size. The cell size control strategy should be robust to different sources of noise such as variation in microenvironment, stochastic gene expression, and heterogeneity in cell physiology and growth. These strategies and their molecular mechanisms are now beginning to be investigated in detail at the single cell level, especially with model bacterial species such as *Escherichia coil* and *Caulobacter crescentus* (Campos et al., 2014; Taheri-Araghi et al., 2015) but how these principles apply to other bacterial systems such as mycobacteria is less well understood.

*Mycobacterium* includes the highly successful pathogen *M. tuberculosis*, the causative agent of tuberculosis, which currently holds the dubious distinction of being the leading cause of death by any single infectious agent, with an estimated 1.8 million deaths worldwide in 2015 (World Health Organisation, 2016). Understanding how mycobacteria control their cell size and decide to divide may be of critical importance in developing novel treatment strategies, especially given the growing problem of drug resistance.

Three basic strategies of cell size control have been proposed, often termed “sizer,” “timer,” and “adder” (Jun and Taheri-Araghi, 2015). These different cell size control strategies can be all described by a single model: a noisy linear map (Jun and Taheri-Araghi, 2015; Tanouchi et al., 2015) that relates division size to birth size:

(1) $$\begin{array}{r} L_{d}=a\cdot L_{b}+b+\eta \end{array}$$

where *L*_*d*_ is cell length at division, *L*_*b*_ is cell length at birth, *a* and *b* describe the slope and intercept of the linear relationship between the two, and η is a noise term that models biological variability in size control. *a* and *b* can be estimated by performing linear regression on *L*_*b*_ against *L*_*d*_, where the slope of the line is *a* and the *y*-intercept is *b*. A slope of *a* = 0 implies that cells follow a sizer model, where every division occurs at a characteristic length *b* irrespective of birth length, providing a simple conceptual method to maintain cell size, since any deviation is immediately reset at every cell division. A slope of *a* = 1 implies an adder model, where cells grow by a fixed length *b* every cell cycle before dividing, which implies convergence to the mean size at birth over multiple generations, since cells that are born small will produce larger daughters, and cells that are born large will produce smaller daughters. A slope of *a* = 2 implies a timer model, where cells divide after a fixed time interval, which necessitates a doubling in cell size every cell cycle to maintain a stable size distribution, though a pure timer mechanism for exponentially growing cells is not robust to noise (Iyer-Biswas et al., 2014).

Timer and sizer models, at least in their purest sense, are not well supported by existing evidence in the majority of bacterial species, with the adder model being strongly favored in gram negative species such as *E. coli* (Amir, 2014; Campos et al., 2014; Taheri-Araghi et al., 2015) and *C. crescentus* (Campos et al., 2014), in gram positive species such as *Bacillus subtilis* (Taheri-Araghi et al., 2015), and more recently in coccoid species such as *Synechocystis* sp. (Yu et al., 2017). Mechanistically, how adder size control is implemented is not completely clear. Establishing how bacteria make this decision has been investigated at the population level (Cooper and Helmstetter, 1968; Cooper, 1969; Helmstetter, 1969) and the single cell level (Wallden et al., 2016; Si et al., 2017) with the conclusion that—at least in *E. coli*—replication is initiated at a given ratio of origins to cell size with the duration of DNA replication being constant, and that the cell divides at a fixed time after replication initiation.

However, mycobacteria have key differences in how they grow and divide. Placement of the septum in *E. coli* is highly accurate (Trueba, 1982; Yu and Margolin, 1999) compared to mycobacteria (Thanky et al., 2007; Joyce et al., 2012; Singh et al., 2013), with the direct consequence that cell size distributions are highly heterogeneous within mycobacterial populations. In addition, while *E. coli* incorporates cell wall material along its lateral wall, mycobacteria grow exclusively from their poles (Thanky et al., 2007), and mounting evidence points to preferential incorporation at a single pole, which is formed in the previous generation (Joyce et al., 2012; Santi et al., 2013; Manina et al., 2015), referred to here as the old-pole. This inheritance of an active growth region by one daughter cell leads to additional heterogeneity between daughter cells especially in parameters such as cell size. *C. crescentus* also divides in an asymmetric manner, giving rise to stalked and swarmer cells which have markedly different life cycles and growth rates, but maintain the adder principle (Campos et al., 2014; Iyer-Biswas et al., 2014) thus asymmetry does not rule out adder models *per se* as other authors have noted. Indeed, in mycobacteria and *M. smegmatis* specifically, there is little evidence for a timer or sizer model, though an adder model is not fully established: Aldridge et al. (2012) use time-lapse microscopy to rule out a sizer model in *M. smegmatis*, but correlate cell cycle timings within microcolonies to suggest a timer model (though the authors reported this prior to widespread publication of the adder model) as well as determining that cells that inherit the old-pole from their mother are larger and have a faster linear elongation rate than cells that inherit the new-pole. Using similar techniques, Santi et al. (2013) determine that *M. smegmatis* are neither sizers nor timers, but observe a weak correlation between added length and length at birth which implies more timer-like behavior. Furthermore, Santi et al. (2013) report that large heterogeneities rapidly emerge as a consequence of preferential polar growth, such as the simultaneous larger size and faster linear elongation rate of daughter cells inheriting the old-pole compared to those inheriting the new-pole, in agreement with Aldridge et al. (2012), but observe no difference in exponential growth rate.

The differences in growth between sister mycobacterial cells are not simple quirks of bacterial growth, but may have a significant impact on bacterial survival and therapeutic strategy. Importantly, differences in exponential growth rate are observed between new- and old-pole inheritors in a recent study on *M. tuberculosis*, with cells inheriting the new-pole growing fractionally faster than siblings inheriting the old-pole (Manina et al., 2015). Pole age has been reported to have a major effect on survival to various antibiotics, with old-pole inheritors more able to survive rifampicin (Aldridge et al., 2012; Richardson et al., 2016), and new-pole inheritors potentially more able to survive cell wall-targeting drugs such as meropenem, cycloserine and isoniazid (Aldridge et al., 2012), though differences in both pole age and growth rate have been reported to have no impact on isoniazid survival by other authors, both in *M. smegmatis* (Wakamoto et al., 2013), and in *M. tuberculosis* (Manina et al., 2015). Given that treatment of *M. tuberculosis* requires at least 6 months of daily antibiotic treatment, factors that affect drug tolerance must be determined, and current evidence may point to a role of cell size control.

The studies discussed above have focused exclusively on bacteria grown with standard laboratory mediums, supplying glycerol or glucose as the sole carbon source. However, mycobacterial physiology is thought to be dramatically altered by carbon source resulting in changes to virulence and dormancy (Jain et al., 2007). *M. tuberculosis* is thought to utilize cholesterol as a primary carbon source *in vivo* (Salamon et al., 2014; Vromman, 2014), causing extensive changes in growth. In this report, we have sought to determine definitively the growth model which mycobacteria follow using time-lapse microscopy coupled with microfluidics to allow tracking of single *M. smegmatis* cell lineages over time, with precise control of environmental conditions, under three carbon sources: glycerol, acetate, and pyruvate, and have sought to determine how this affects cell size control and population heterogeneity at the single-cell level.

## 2. Materials and methods

### 2.1. Strains and mediums

*M. smegmatis* mc^2^ 155 (Snapper et al., 1990) was used in all experiments, and was grown in medium derived from Hartmans-de Bont minimal medium (HdB; Hartmans and De Bont, 1992) with a base composition as previously described (Smeulders et al., 1999), with the replacement of 0.05% Tween-80 with 0.05% (v/v) tyloxapol (Sigma), a non-hydrolysable alternative detergent. In addition, for experiments involving alternative carbon sources, glycerol was replaced with either 0.2% (w/v) potassium acetate (Sigma) or 0.2% (w/v) sodium pyruvate (Sigma). Middlebrook 7H11 agar (Difco) supplemented with 10% OADC (USBiological) was used for solid medium experiments.

### 2.2. Batch culture experiments

For batch culture growth experiments, cells were inoculated into their HdB carbon source variant from frozen glycerol stocks and grown at 37 °C in an orbital shaker at 180 rpm for 48 h until cultures reached stationary phase. Cultures were diluted to an optical density at 600 nm (OD_600_) of 0.05 in 20 ml fresh medium and incubated at 37 °C in an orbital shaker at 180 rpm. Samples were taken each day at 2–4 h intervals over a 4 day period. OD_600_ was measured in a spectrophotometer, and each sample was subsequently serially diluted and spotted onto 7H11 agar plates, which were incubated for 48 h until visible distinct colonies formed. Colony forming units (CFUs) were counted and CFU/ml calculated.

### 2.3. Microscopy

All microscopy was performed in the Facility for Imaging by Light Microscopy (FILM) at Imperial College London. Time-lapse live-cell microscopy was performed using a CellASIC® ONIX microfluidic platform (Merck-Millipore) using a B04A-03 bacterial plate. Cells were inoculated from glycerol stocks in their appropriate HdB carbon source variant and grown at 37 °C in an orbital shaker at 180 rpm to stationary phase, then diluted 1:200 in fresh medium to mid-exponential phase. Cells were diluted again to an OD_600_ of 0.1 and loaded into the growth chamber of the microfluidic plate. Fresh medium was perfused at a continuous pressure of 1 psi (approximately corresponding to a 5 μl h-1 flow rate) in an environmental chamber at 37 °C. Phase contrast images were captured every 15 min using a Zeiss Axiovert 200M inverted widefield microscope using a 63X oil immersion objective, a motorized stage, and an EM-CCD (C9100-02) camera (Hamamatsu). 4 fields of view per condition (4 conditions per plate) were captured at each timepoint. To ensure in-focus images were obtained, a software autofocus function (Zen) and 10 Z slices at 1 μm intervals were used at each position.

### 2.4. Data analysis

#### 2.4.1. Image processing

Raw microscopy data were processed using Fiji imaging processing software (Schindelin et al., 2012) to discard unfocused images and to generate time-lapse sequences in an appropriate file format (OME-TIFF). Cells in each frame were outlined in a semi-automated manner with MicrobeTracker, a MATLAB (The Mathworks) software package that detects bacterial cell boundaries and delineates them using a two-dimensional mesh (Sliusarenko et al., 2011). Cell division was defined as the point at which clear invagination of the cell or V-snapping could be observed. Single-cells detected by MicrobeTracker were arranged into lineages and analyzed using an automated set of Python scripts, with manual verification of assignments. For each cell, the following basic statistics were defined: cell length, the distance along the central axis of the cell between each cell pole; and pole age, the incremented value derived from whether a pole is pre-existing in the mother cell (old-pole), or created at division (new-pole). Old-pole inheritance was determined by calculating the distance between both poles of a daughter cell to both poles of the mother cell, and classing the pair with the smallest distance as the same pole and therefore the old pole. Birth and division times, as well as initial and final cell lengths at these timepoints were also recorded, but were refined by interpolation as defined below. For selection of initial and final cell lengths, any cell in which clear birth or clear division event could not be observed were discarded (i.e., cells in the first and last frames) and any cell that was not observed over a period of at least 5 frames was also discarded. Independent experiments were performed in triplicate for cells grown in glycerol, and in duplicate for cells grown in acetate or pyruvate.

#### 2.4.2. Growth rate estimation and model selection

Growth rates were estimated from time-series of length *L*_1_, …, *L*_*n*_ observed in discrete frames at times *t*_1_, …, *t*_*n*_. Linear elongation rate was determined by linear regression of cell length against time (*L*(*t*) = γ·(*t*−*t*_1_) + *L*_*I*_), and exponential growth rate was estimated from regression of logarithmic cell length against time (ln(*L*(*t*)) = γ · (*t* − *t*_1_) + ln(*L*_*I*_)).

To decide whether cells grow exponentially or in a linear fashion we performed model selection. We describe cell growth via the dynamical equation $\frac{d}{dt}L=\gamma f\left(L\right)+\sigma \epsilon \left(t\right)$, where ϵ(*t*) is Gaussian white noise with unit variance, *f*(*L*) = 1 for the linear and *f*(*L*) = *L* for exponential growth model. Discretising the paths in time-steps of δ*t*_*i*_ = *t*_*i*+1_ − *t*_*i*_, the path-likelihood for either model is:

$$\begin{array}{r} L\left(\hat{\gamma },{\hat{\sigma }}^{2}|\left\{L_{1},\ldots ,L_{n}\right\}\right)=\overset{n-1}{\underset{i=1}{\prod }}\frac{e^{-\frac{{\left(L_{i+1}-L_{i}-\delta t_{i}\hat{\gamma }f\left(L_{i}\right)\right)}^{2}}{2\delta t_{i}{\hat{\sigma }}^{2}}}}{{\left(2\pi {\hat{\sigma }}^{2}\delta t_{i}\right)}^{1/2}}, \end{array}$$

whose maximum likelihood estimates are $\hat{\gamma }=\overset{n-1}{\underset{i=1}{\sum }}\delta t_{i}\left(L_{i+1}-L_{i}\right)f\left(L_{i}\right)/\left(\overset{n-1}{\underset{i=1}{\sum }}\delta t_{i}f^{2}\left(L_{i}\right)\right)$ and ${\hat{\sigma }}^{2}=\frac{1}{n-1}\overset{n-1}{\underset{i=1}{\sum }}\delta t_{i}{\left(\frac{L_{i+1}-L_{i}}{\delta t_{i}}-\hat{\gamma }f\left(L_{i}\right)\right)}^{2}$ for each cell. According to Akaike's information criterion, the growth model with the highest likelihood was selected.

#### 2.4.3. Estimation of cell division time, birth length and division length

The precise time at which cell division occurs remains unknown, because images were acquired at a constant time interval. To estimate this quantity, we used the exponential model for all cells and minimized numerically the absolute difference between the mother cell length and length of the two daughter cells |*L*_*m*_(*t*) − *L*_1_(*t*)−*L*_2_(*t*)| over the time interval between mother and daughter frames, where the functions *L*_*i*_(*t*) are the fits obtained from linear regression to log-cell length as explained above. The argument of the minimum gives the division time, and division length of the mother cell and birth lengths of the two daughters were set to the values at that time. All subsequent analysis uses birth length and division length as estimated by this procedure (as opposed to initial length and final length as defined above), and interdivision time defined as the difference between these two interpolated timepoints.

#### 2.4.4. Population and lineage-weighted statistics

Population statistics were obtained from averaging all cells in the experiments with equal weights. If not stated otherwise, error bars were obtained with ± reference the standard 95% confidence interval. Lineage-weighted statistics were obtained by reconstructing the lineage trees from microscopy data. Denoting by *x*_*ij*_ the data of the *i*^*th*^ of *N*_*j*_ cells in the *j*^*th*^ of *N*_*L*_ lineages, the lineage-weighted average of the function *f* is:

$$\begin{array}{r} \overset{®}{f}=\overset{N_{L}}{\underset{j=1}{\sum }}\overset{N_{j}}{\underset{i=1}{\sum }}w_{j}f\left(x_{ij}\right). \end{array}$$

The weights *w*_*j*_ are given by $\frac{2^{-D_{j}}}{N_{T}N_{j}}$, where *D*_*j*_ denotes the number of cell divisions in lineage *j*. They follow from the argument that the probability to choose a random path through *N*_*T*_ trees is $2^{-D_{j}}/N_{T}$ (Nozoe et al., 2017). We estimate the lineage-weighted distribution by choosing *f* to be an appropriate binning function. Bootstrap confidence intervals reported in Table S1 are obtained by re-sampling the data *x*_*ij*_ with probability given by the lineage-weights *w*_*j*_.

## 3. Results

### 3.1. *M. smegmatis* grows exponentially at the single-cell level

**Figure 1:**
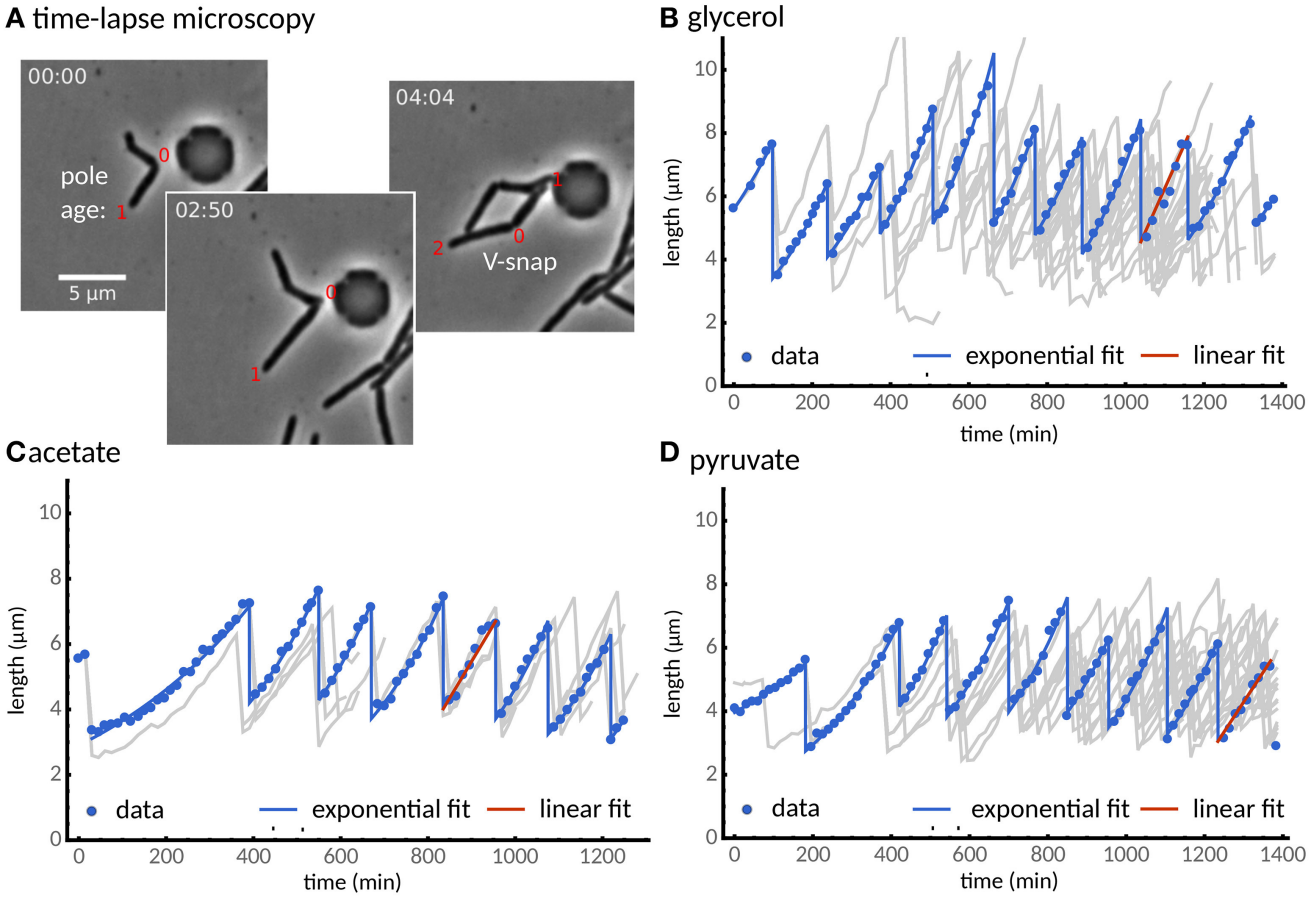
Single cells grow exponentially under different carbon sources. **(A)** Time-lapse microscopy shows single cells grown with a glycerol carbon source observed from cell birth to division determined by the V-snap (from left to right). **(B)** Cell length vs. time for one of the longest lineage of the experiments (points). The lineage tree arising from all recorded cell divisions is shown in gray. **(C,D)** Two alternative carbon sources, **(C)** acetate and **(D)** pyruvate, are considered. Under all three carbon sources, most cells (about 90%) grow exponentially (blue lines), while some cells were better described by a linear growth model (red line). We observed that cells adapted to the experimental conditions after about two divisions.

Figure 1 shows cell size vs. time for examples of cell lineages obtained from the 3 different growth conditions. In the literature, different measures have been used to quantity the growth of single mycobacteria. Among these linear growth rate (e.g., Aldridge et al., 2012) and exponential growth rate (e.g., Wakamoto et al., 2013) are predominant but have led to qualitatively different conclusions. Other authors have reported that elongation in single-cells is size-dependent (Santi et al., 2013) indicating exponential growth. Indeed, we find that on average the increase in cell length scales linearly with cell length for all three growth media (Figure S1). To address this issue on the single-cell level, we fitted a linear and an exponential growth model of cell length to each cell, and employed model selection to decide which of these models more adequately describes cell growth (see Materials and Methods). We find that for cells grown in glycerol, acetate, and pyruvate, growth is also exponential at the single-cell level for about 90% of the analyzed cells, independent of carbon source (Figure 1). Because we observed cells adapting to our experimental conditions (Figures 1C,D), cells are included in the subsequent analysis only after they divided twice in the microfluidic device. We note that we observe adaptation in all conditions, though to a lesser extent in glycerol, consistent with CFU measurements in batch culture (Figure S6B).

### 3.2. Under standard conditions, *M. smegmatis* single cells are consistent with an adder model of cell size control

**Figure 2:**
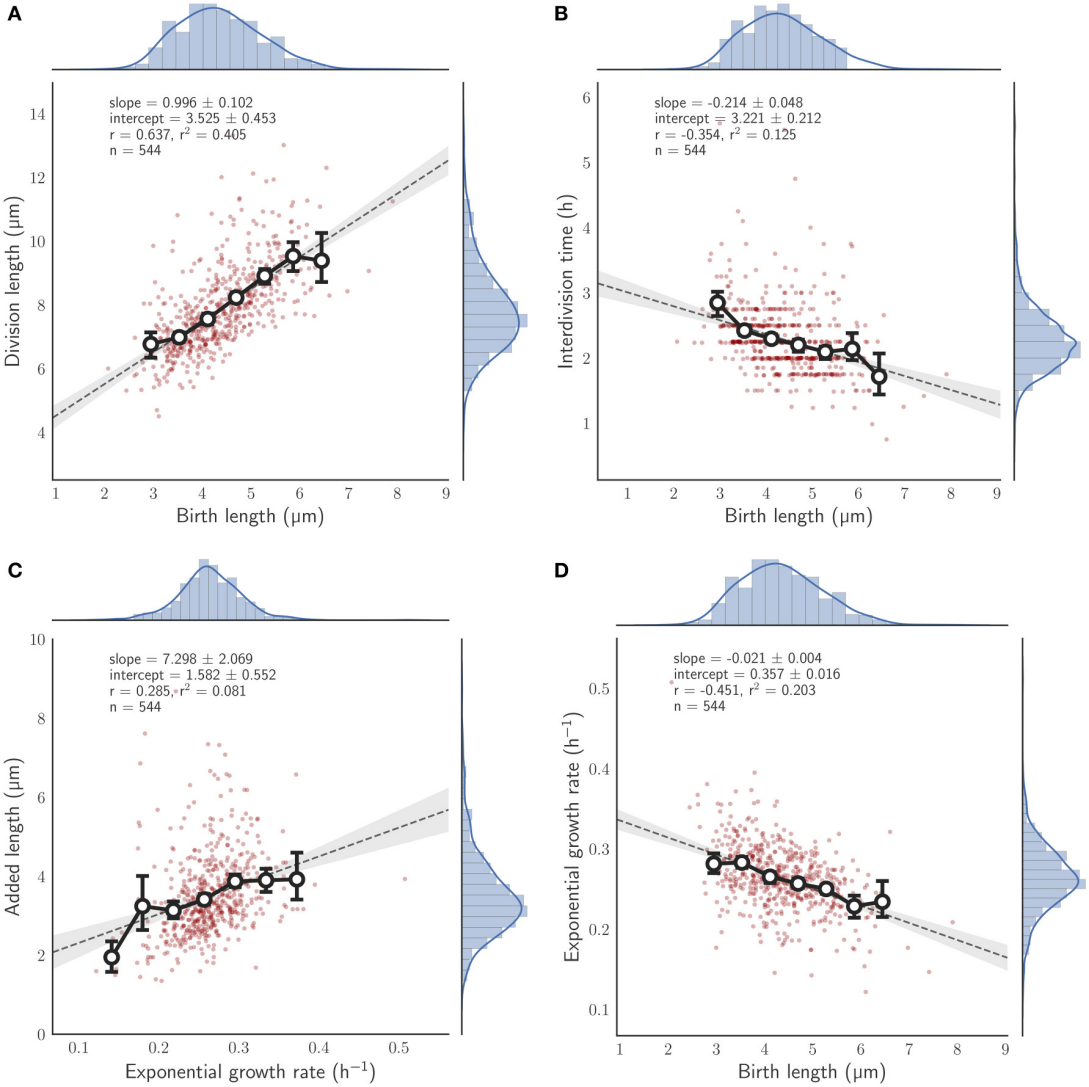
Relationship between birth length and other single cell measures for single *M. smegmatis* cells grown with glycerol as a sole carbon source. Red circles, single cell measurements; histograms, distribution of individual cells, with kernel density estimates depicted as a blue line; solid line with white filled circles, mean of birth length bins with error bars denoting the 95% confidence interval of the estimate determined by bootstrapping; gray dotted line, the least squares linear regression fit of unbinned data; Key, *slope*—the slope of the regression line (± the 95% confidence interval), *intercept*—the *y*-intercept, *r*—the Pearson correlation coefficient, *n*—the total number of cells measured, **(A)** Division length is plotted against birth length. **(B)** Interdivision time against birth length. **(C)** Added length against exponential growth rate. **(D)** Exponential growth rate against birth length.

We observed 544 individual *M. smegmatis* cell divisions, in three independent experiments, grown using HdB supplemented with glycerol as a sole carbon source, measuring cell length at birth and at division over up to 10 generations (see Section 2). A linear regression to these data according to Equation (1) results in *a* = 0.996 ± 0.102, *b* = 3.525 ± 0.453 μm (Figure 2A). These values imply that *M. smegmatis* cells closely follow an adder model of cell size control when grown with glycerol as a sole carbon source, with cells growing an average of 3.5 μm per cell cycle, independent of birth size. Alternatively, cell length at birth can be fitted to added length (defined as the difference in length at birth and division) which results in a slope of −0.004 ± 0.102 and a Pearson's *r*-value of −0.003, strongly indicating that added length does not depend on length at birth (Figure S4C1). This lack of dependency on birth length has been interpreted by other authors (e.g., Aldridge et al., 2012) as evidence that *M. smegmatis* cells follow a timer mechanism. A timer model requires a fixed interdivision time for all cells irrespective of length at birth. However, when grown with glycerol as a sole carbon source, interdivision time is negatively correlated with birth length (Pearson's *r* = −0.354; Figure 2B), demonstrating that cells that are born small grow for a longer period than cells that are born large, which is inconsistent with the timer model.

Other authors have reported synchronization of interdivision times within microcolonies (cell lineage trees founded by a single cell) that are characteristic of that microcolony and can differ significantly between microcolonies (Aldridge et al., 2012). In order to address whether this occurs under our conditions, we selected random lineage trees with 4 or more observed divisions and determined how their interdivision times compared. While inspection of individual microcolonies appears to indicate that microcolony interdivision times might be synchronized (Figures S2A,B), mean interdivision times appear to have no significant differences between microcolonies (*p* = 0.520; Kruskal-Wallis H-test) indicating that cells simply conform to the population mean interdivision time without inheritance from the progenitor cell (Figure S2C).

While these results indicate that *M. smegmatis* cells closely follow an adder model under these conditions, we asked what other factors affect size control. We found that added length increases with exponential growth rate (*r* = 0.285; Figure 2C). Thus, growth rate fluctuations in individual cells can have considerable effects on size control even under constant conditions. Interestingly, we also found that exponential growth rate decreases with birth length (*r* = −0.451; Figure 2D) at least when grown in glycerol (compare Figure S4D1); a dependency that is not observed in *E. coli* (Campos et al., 2014; Osella et al., 2014; Taheri-Araghi et al., 2015) or in *C. crescentus* (Campos et al., 2014). Since *M. smegmatis* divide asymmetrically (Aldridge et al., 2012; Joyce et al., 2012; Manina et al., 2015), birth lengths are inherently variable, and thus this dependence hints at variable growth rates between daughter cells.

### 3.3. Asymmetry in division and pole age has no impact on cell size control under standard conditions

**Figure 3:**
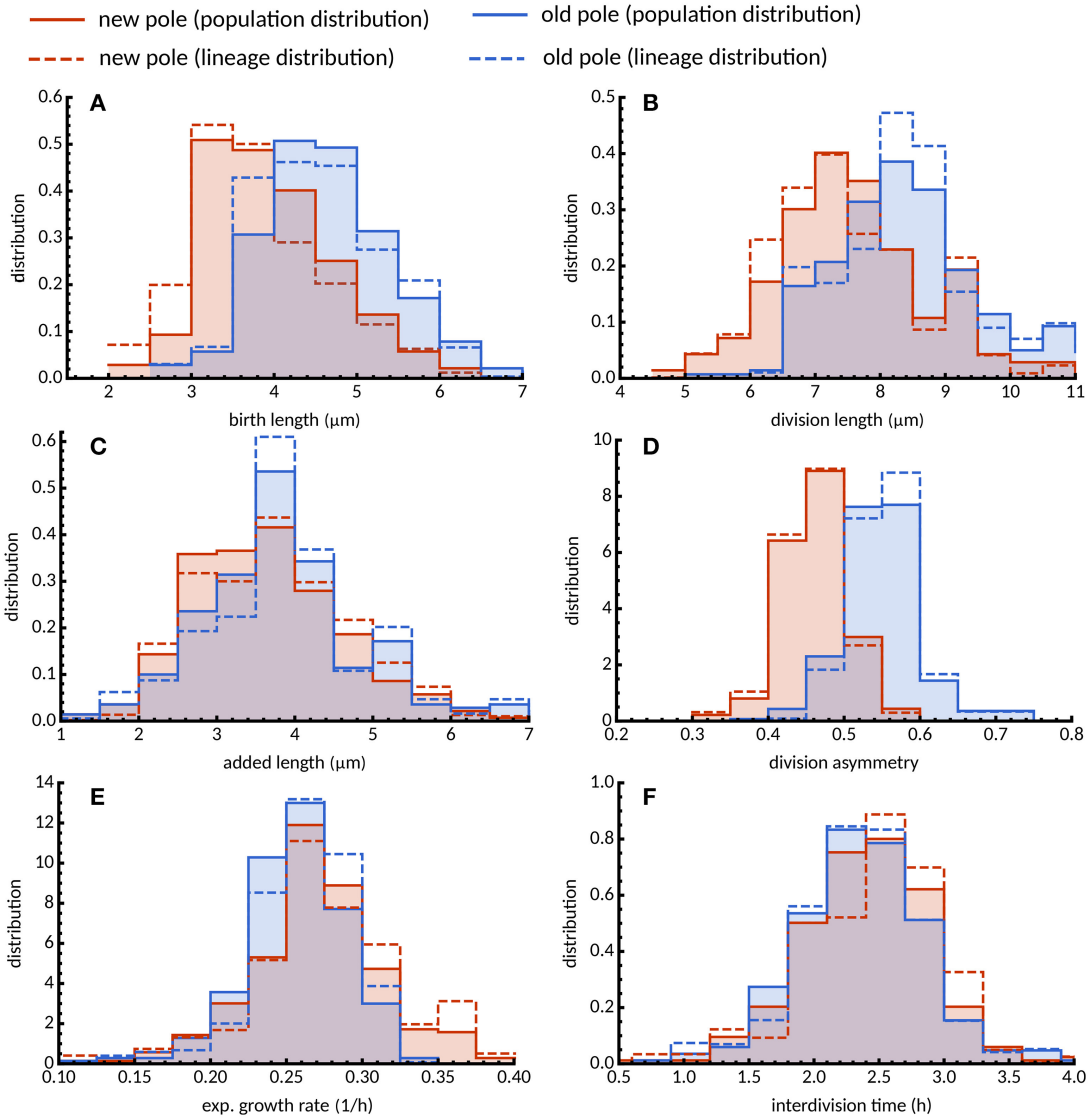
Distribution of growth parameters for new- and old-pole cells grown in glycerol. Histograms of **(A)** birth length, **(B)** division length, **(C)** added length, **(D)** division asymmetry, **(E)** exponential growth rate, and **(F)** interdivision time are shown for new (red) and old pole cells (blue). Population average distributions taken over every single cell in the experiment (solid lines) are qualitatively similar compared to lineage-weighted distributions (dashed lines).

**Figure 4:**
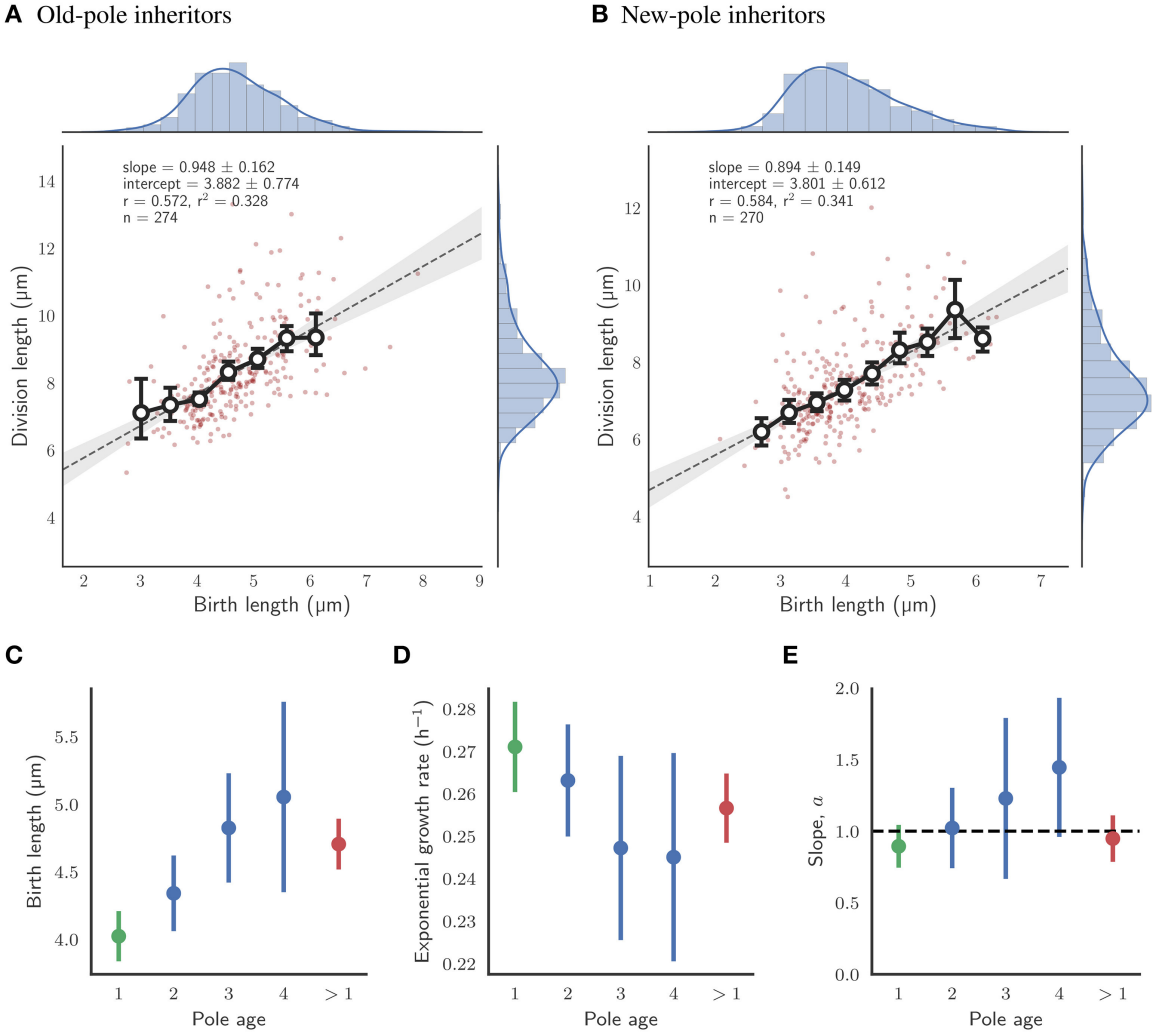
Impact of pole age on the growth of single cells with glycerol as a sole carbon source. **(A)** Fit of division length against birth length for daughter cells which inherit the old-pole from their mother cell. Colors and legends as for Figure 2. **(B)** Fit of division length against birth length for daughter cells which inherit the new-pole from their mother cell. Colors and legends as for Figure 2. **(C)** Mean birth length for cells by age of the inherited pole in number of generations. **(D)** Mean exponential growth rate by age of the inherited pole. **(E)** Slope of the linear fit of birth length against division length for cells by age of the inherited pole. The black dashed line represents the case where cells act as perfect adders (*a* = 1). **(C–E)** The error bars depict the 95% confidence intervals of each estimate. *n*-values were 99, 39, and 21 in order of generation number for old-pole cells with known age (shown in blue). Data for all new-pole inheritors (with an age of 1 generation; *n* = 270), and for all old-pole inheritors (with an age of more than 1 generation, including those without a precise age; *n* = 272) are shown in green and red respectively.

The implications of asymmetry in division on cell size control have not been investigated in detail, and the time-lapse data were used to address this question. Cells which were observed to divide during the course of the experiment were assigned a pole age based on the history of the cell lineage, allowing daughters which inherited the old- or new-pole from their mother to be differentiated. As previously reported (Aldridge et al., 2012; Joyce et al., 2012; Santi et al., 2013), we observe marked differences in birth and division length, and linear and exponential growth rate for each type of daughter cell (Figure 3). Cells inheriting the old-pole were born larger (4.71 ± 0.19 μm) and divided larger (8.34 ± 0.31 μm) than cells inheriting the new-pole (4.02 ± 0.19 μm and 7.40 ± 0.28 μm respectively). Notably, new-pole inheritors have a higher exponential growth rate than old-pole inheritors (0.271 ± 0.110 h-1 and 0.257 ± 0.800 h-1 respectively), in agreement with recent single-cell data acquired from *M. tuberculosis* (Manina et al., 2015) but in contrast to reports of identical exponential growth rates in *M. smegmatis* single cells (Santi et al., 2013; Wakamoto et al., 2013). Interestingly there was also a significant difference in added length (3.64 ± 0.26 and 3.38 ± 0.23 μm for old- and new-pole cells respectively; *p* = 0.003; Welch's *t*-test) but not in interdivision time (2.27 ± 0.12 and 2.31 ± 0.13 h respectively; *p* = 0.393; Welch's *t*-test). The impact of these differences in growth parameters were assessed by determining the dependence of division length on birth length. Linear regression showed that daughters that inherited the old-pole had a slope of 0.948 ± 0.162) (*n* = 274; Figure 4A), and daughters that inherited the new-pole had a slope of 0.894 ± 0.149) (*n* = 270; Figure 4B). Both these values are consistent with an adder model of cell size control (*a* = 1) and are not significantly different (*p* = 0.481; Welch's *t*-test). These data imply that daughter cells inheriting the old (and presumably growing) pole follow the same cell size control mechanism compared to daughter cells inheriting the new-pole, despite differences in their growth.

Statistics of tree-structured data can be biased when, due to physiological fluctuations, some cells are over-represented in the population because they divide more rapidly than others (Powell, 1956; Hashimoto et al., 2016; Thomas, 2017). To reduce this biological bias, we use lineage-weighting of the data, which represents the statistics obtained from randomly chosen lineages (see Materials and Methods). For cells grown in glycerol, interdivision time was lower across the population compared to lineages for both new- and old-pole cells (Figure 3F) indicating that cells that divide faster are contributing more to the population mean (2.485 and 2.756 h, respectively; Table S1). Intuitively, one would expect other factors affecting division timings such as exponential growth rates to be similarly skewed (Thomas, 2017). However, we observe only small differences between population and lineages both in terms of mean exponential growth rate (Table S1) and distributions (Figure 3E). This can be explained by the fact that interdivision time varies little with exponential growth rate for cells grown in glycerol (Figures S4F2, S5F2), a surprising result given that cells with constant added length should divide earlier when growing faster. In support of this observation is the dependence of added length on exponential growth rate (Figure 2C), with cells growing faster adding more length before division. Since the overall differences between population and lineage distributions were relatively small (Figure 3E), they do not appear to affect conclusions drawn from population statistics.

In order to investigate further whether pole age might affect size control, old-pole inheritors were subdivided by their definitively known pole age—i.e., the pole was observed to be “born” as a new-pole, and tracked over multiple generations to assign an age. Statistics such as birth and division length, and linear elongation rate appear to progressively increase with pole age (Figures S5A5, B5, E5); exponential growth rate marginally reduces (Figure S5D5); and added length and interdivision time appear to remain constant (Figures 4C,D; Figures S5C5, F5). Consistent with the grouped data, though *n*-values are low for higher pole ages, there appears to be no evidence that the age of the inherited pole causes deviation from the adder model, with the slope of birth length (*a*) against division length ranging 1.022 ± 0.281 for two generation-old poles (*n* = 99) to 1.446 ± 0.486 for four generation-old poles (*n* = 21) which are within the range predicted by perfect adders, where *a* = 1 (Figure 4E). A dependence of birth length on pole age (Figure 4C) is a direct prediction of the adder principle given asymmetry conferred by inheritance of the old pole. Old-pole inheritors are born larger (Figure 3) than new-pole siblings, but as a consequence of the adder principle, the same length is added regardless of birth length, thus as pole age increases, birth length increases.

### 3.4. Changing carbon source causes deviation from adder

HdB contains glycerol as the sole carbon source (see Materials and Methods) in which *M. smegmatis* is able to grow efficiently and rapidly. However, this is unlikely to represent conditions under which bacteria habitually live, *M. tuberculosis* for example is thought to catabolize cholesterol as its primary carbon source during human infection (Salamon et al., 2014; Vromman, 2014). In order to investigate how mycobacteria control their cell size under less optimal carbon sources, *M. smegmatis* cells were grown with acetate or pyruvate as their sole carbon source, two of the metabolic products of cholesterol metabolism. Cells grown in batch culture with acetate and pyruvate display reduced growth rates compared to cells grown in glycerol as measured by optical density (Figure S6A) and colony-forming units (Figure S6B). These population level measures are consistent at the single-cell level, with cells grown in the microfluidic device showing reduced linear elongation rates and increased interdivision time (Table 1).

**Table 1:** Single-cell growth parameters for *M. smegmatis* cells grown in various single carbon sources in a microfluidic device.

**Carbon source**	***n***	**Birth length (μm)**	**Interdivision time (h)**	**Exponential growth rate (h^−1^)**	**Division asymmetry**
Glycerol	544	4.367 ± 0.144	2.286 ± 0.087	0.264 ± 0.007	0.555 ± 0.016
Acetate	383	3.914 ± 0.112	2.389 ± 0.096	0.265 ± 0.007	0.522 ± 0.013
Pyruvate	367	3.556 ± 0.108	2.726 ± 0.128	0.235 ± 0.007	0.542 ± 0.016

*Division asymmetry here is defined as the asymmetry in old-pole inheritors. Further definitions of all measures can be found in the Materials and Methods*.

**Figure 5:**
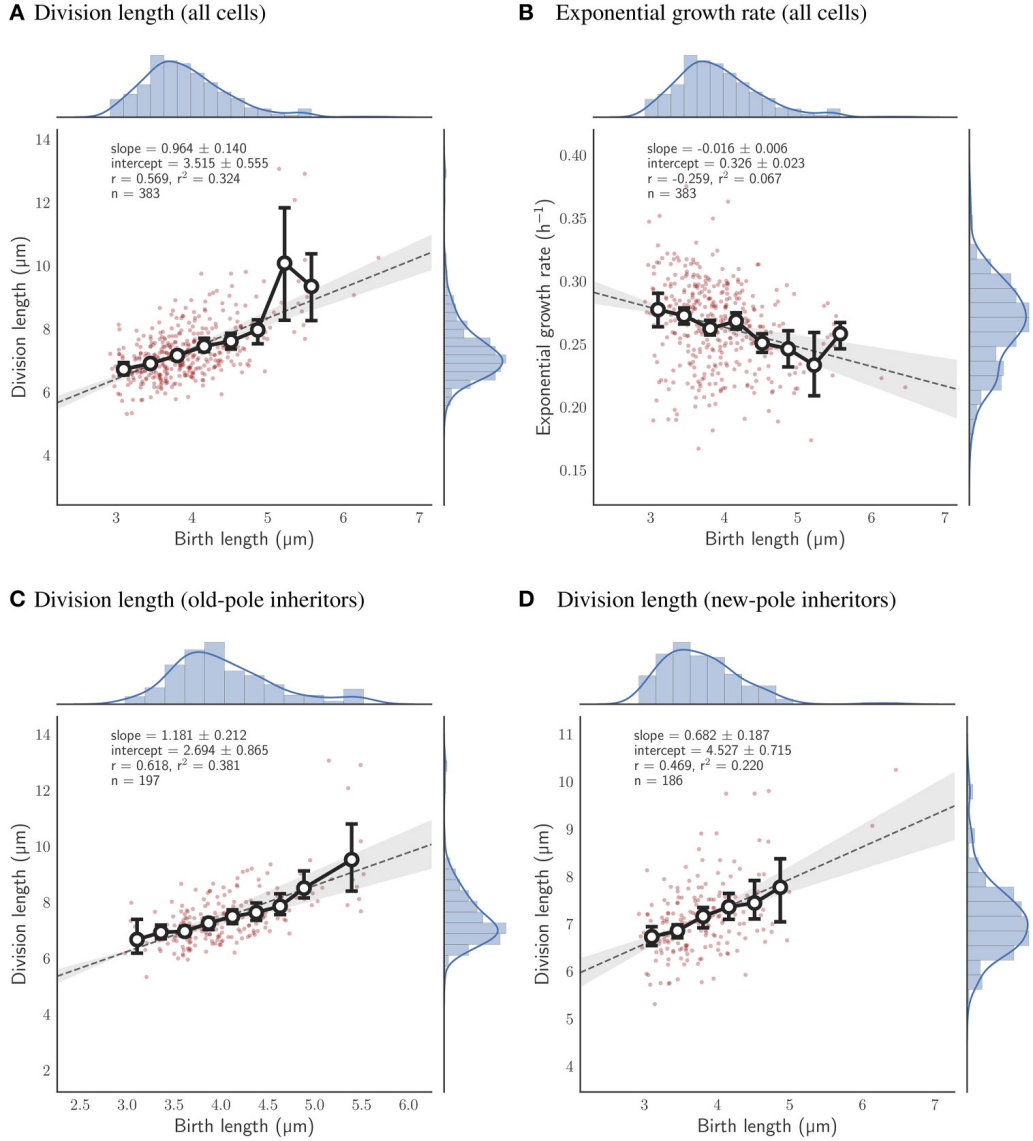
Relationship between birth length and division length or exponential growth rate for cells grown with acetate as a sole carbon source. Colors and legends as for Figure 2. **(A)** Relationship between birth length and division length for all cell divisions. **(B)** Relationship between birth length and exponential growth rate for all cell divisions. **(C)** Relationship between birth length and division length for cells which inherit the old-pole from their mother cell. **(D)** Relationship between birth length and division length for cells which inherit the new-pole from their mother cell.

Given the robustness of the adder principle in *M. smegmatis* cells grown in glycerol, alternative carbon sources appear to have a surprisingly significant effect on cell size control (**Figure 7A**). When grown exclusively with acetate, the slope of division length against birth length is 0.964 ± 0.140 (*n* = 383), consistent with an adder (Figure 5A). However, pole age has an effect on the decision to divide when grown in acetate: cells inheriting the existing pole (old-pole) have a slope of 1.181 ± 0.212 (*n* = 197) for division length against birth length, consistent with an adder model, with a hint of more timer-like behavior (Figure 5C), but cells inheriting the newly formed pole (new-pole) have a significantly reduced slope of 0.682 ± 0.187 (*n* = 186; *p* = 0.0006, Welch's *t*-test) which is not consistent with an adder cell (Figure 5D). This indicates some dependence of added length on birth length (i.e., more sizer-like properties), and indeed birth length and added length are uncorrelated for old-pole inheritors (*r* = 0.119), whereas there is a minor negative correlation for new-pole inheritors (*r* = −0.240), indicating that cells that inherit the new-pole that are born larger add less length per cell cycle than cells inheriting the old-pole (Figure S5).

**Figure 6:**
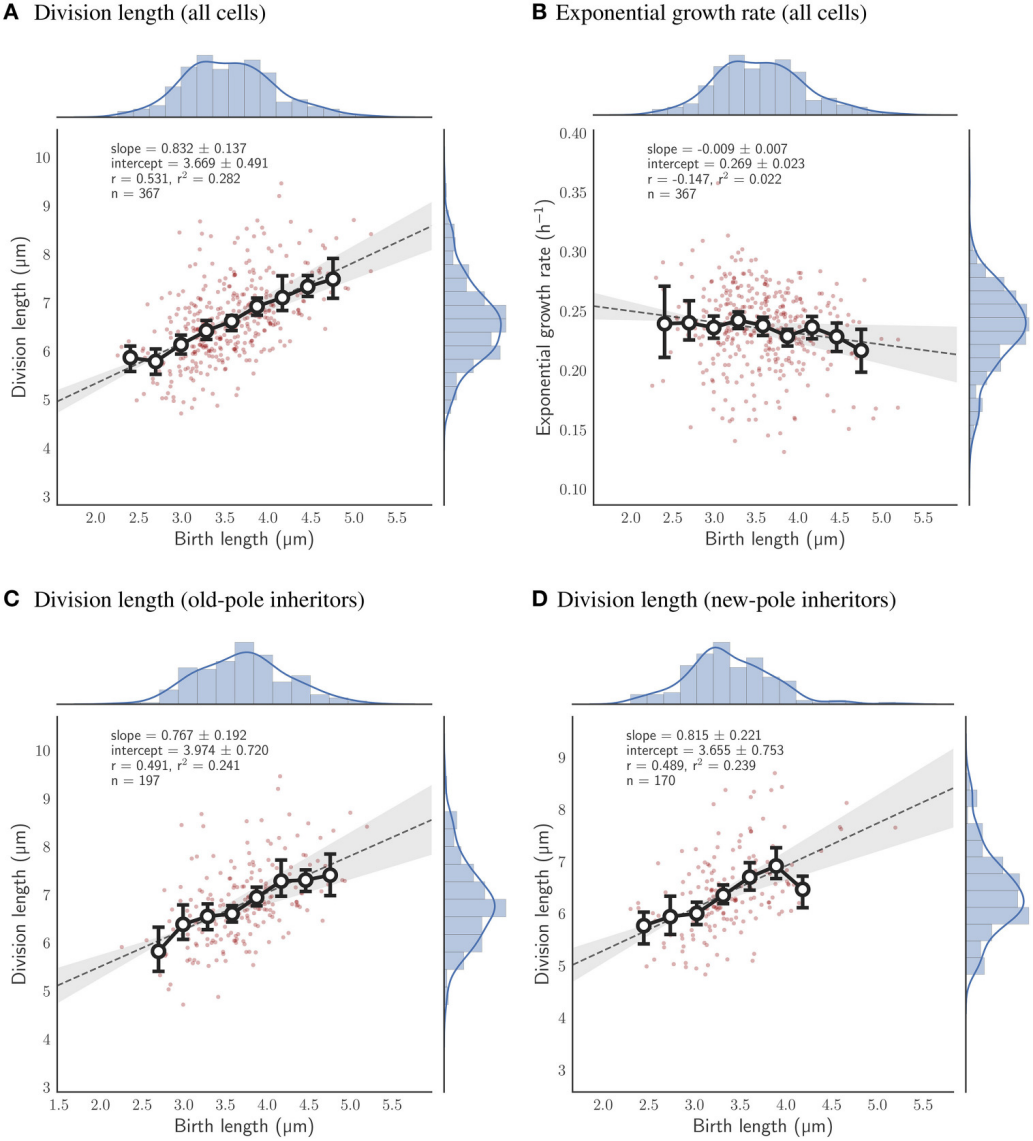
Relationship between birth length and division length or exponential growth rate for cells grown with pyruvate as a sole carbon source. Colors and legends as for Figure 2. **(A)** Relationship between birth length and division length for all cell divisions. **(B)** Relationship between birth length and exponential growth rate for all cell divisions. **(C)** Relationship between birth length and division length for cells which inherit the old-pole from their mother cell. **(D)** Relationship between birth length and division length for cells which inherit the new-pole from their mother cell.

Cells that are grown exclusively in pyruvate deviate as a population from the adder principle regardless of which pole they inherit. The slope of birth length against division length is 0.832 ± 0.137 (*n* = 367; Figure 6A), with old-pole cells and new-pole cells retaining similar slopes: 0.767 ± 0.192 (*n* = 197) and 0.815 ± 0.221 (*n* = 170) respectively (Figures 6C,D). This indicates that cells grown in pyruvate have more sizer-like properties but do not retain differences between old- and new-pole inheritors as observed for cells grown in acetate. This may relate to the more significant reduction in growth observed for cells grown in pyruvate compared to acetate (Table 1), with cells grown in acetate retaining similar single-cell growth characteristics to cells grown in glycerol, whereas cells grown in pyruvate exhibit reduced linear and exponential growth rate, and increased interdivision time (Table 1, Figure S3).

We note that when assessing the differences between population and lineage statistics, interdivision times were smaller for the population measures in all three carbon sources (Figure 3F and Figures S3F1–F3), consistent with the intuition that rapidly dividing cells are over-represented in the population measures. Exponential growth rates in the population were also larger for cells grown in acetate and pyruvate (Figures S3E2, E3), a dependence which is not observed in glycerol (Figure 3E and Figure S3E1). This is because interdivision time decreases with exponential growth rate for cells grown in acetate and pyruvate, i.e., fast growing cells divide faster, but not in glycerol (Figures S4F2, S5F2). The differences in exponential growth rate between population and lineage-weighted statistics appear to be more significant for cells grown in acetate compared to pyruvate (Figure S3E) with the mean statistics showing a decrease from 0.265 h^−1^ [0.261–0.267] (95% confidence interval) to 0.242 h^−1^ [0.241–0.244] in the lineage statistics for cells grown in acetate, compared to 0.235 h^−1^ [0.232–0.239] to 0.223 h^−1^ [0.222–0.225] in pyruvate (Table S1, Figure S3E).

We asked whether other factors could affect cell size control across conditions. We found that birth and added length decreased both in acetate and pyruvate compared to glycerol (Figures S3A,C and Table S1), but growth rate decreased significantly only for pyruvate but not for acetate (Table S1). Division asymmetry between old and new pole cells decreased but to a lesser extent in acetate than in pyruvate (Figure S3D and Table S1). Added length increased with growth rate for all conditions, but growth rates varied less with birth length in acetate and pyruvate than in glycerol (Figures 2D, 5B, 6B and Figure S4C2). The latter dependence can be attributed to decreasing mean growth rates with pole age in glycerol, while growth rates in acetate and pyruvate differ only between old and new pole cells (Figure S4D5).

### 3.5. Impact of carbon source on variability in size control

**Figure 7:**
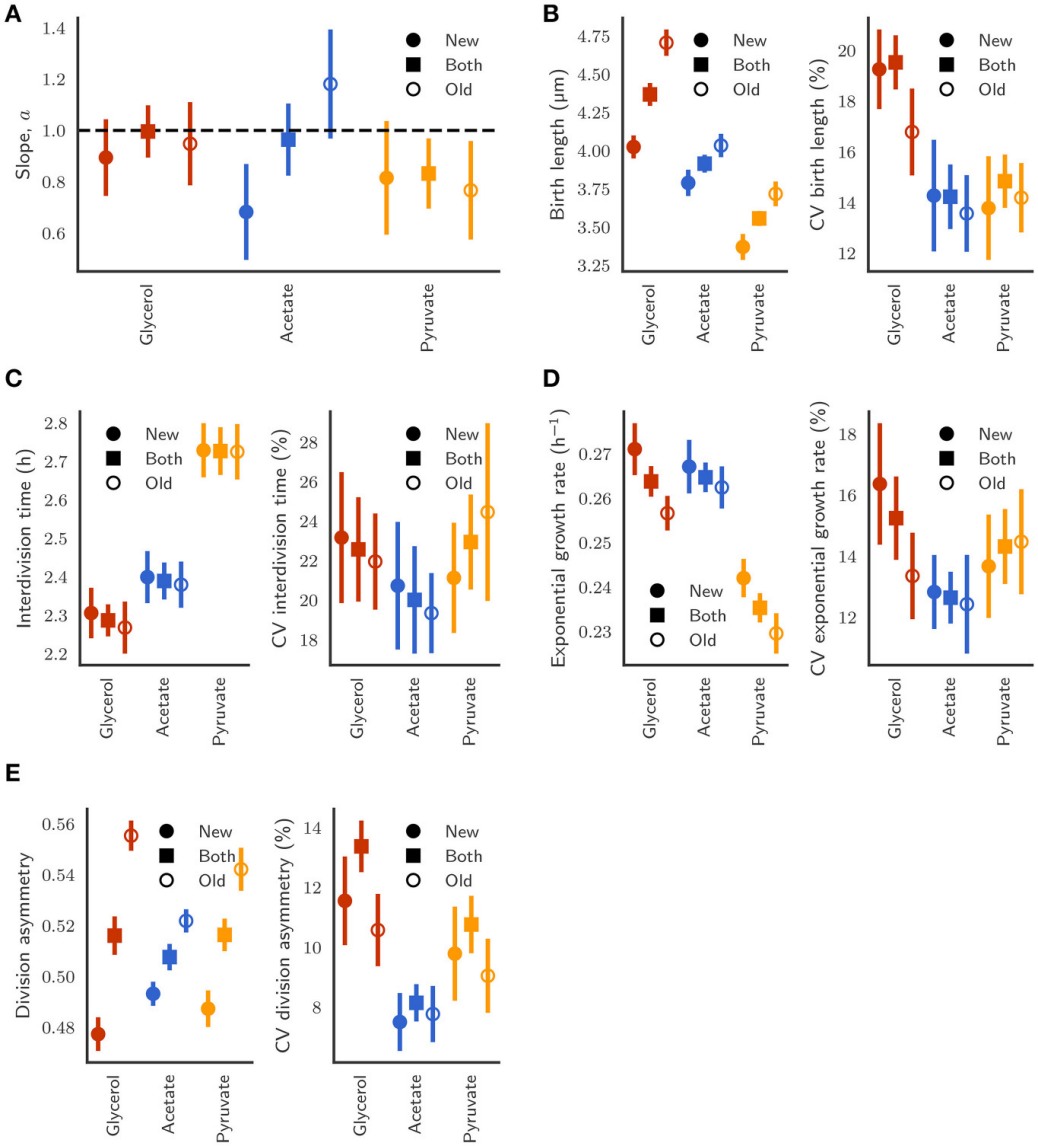
**(A)** Comparison of the slope of the linear relationship between initial length and final length between new-pole (closed circles), old-pole (open circles), and both pole (closed squares) daughter cells. Error bars depict bootstrapped 95% confidence intervals. **(B)** Birth length and coefficient of variation (CV) of the distributions. **(C)** Interdivision time and CV. **(D)** Exponential growth rate and CV. **(E)** Division asymmetry and CV, where asymmetry is defined as the ratio of birth length to the sum of birth lengths of both siblings.

Given differences in the impact of pole inheritance and carbon source on cell size control (Figure 7A), we sought to investigate the consequences of these differences. Other authors have noted that for *E. coli* cells with differing population growth rates under diverse carbon sources, deviations in birth length and interdivision time scale in proportion to their mean value, i.e., distributions under different conditions collapse (Taheri-Araghi et al., 2015; Kennard et al., 2016). We tested whether this holds for *M. smegmatis* grown in glycerol, pyruvate, and acetate, given that, at least between pyruvate and glycerol/acetate, the mean population growth rate is altered (Table 1). We find that distributions for birth length (Figure S7A) do not scale with the mean as demonstrated by the broadness and height of the scaled histograms (Figure S7B), indicating that noise in birth length is altered under different carbon sources. Interdivision time distributions are inherently more noisy, due to the sampling frequency of the image acquisition, and appear significantly right-skewed (Figure S7C), and when scaled by the mean, appear to loosely collapse, as signified by the similar height of the scaled histograms (Figure S7D). This indicates that interdivision time distributions can be represented by a single explanatory variable – the mean value.

Regarding noise for different parameters across growth conditions, we observe reduced variation in birth length for cells grown in acetate or pyruvate compared to cells grown in glycerol with coefficients of variation (CVs) of 19.53% [18.52–20.55] (95% confidence interval), 14.23% [12.73–15.30], and 14.84% [13.89–15.79] for cells grown in glycerol, acetate, or pyruvate respectively (Figure 7B), a result that is reflected visually by the width of the scaled histograms in Figure S7B. The collapse of interdivision time distributions observed (Figure S7D) is also reflected in the similar CVs with 22.57% [19.93–25.39], 20.01% [17.29–21.76], and 22.94% [20.54–25.20] for glycerol, acetate, and pyruvate respectively (Figure 7C).

When pole age is considered, relatively similar variability is observed between daughters inheriting differently aged poles, even given marked differences in absolute mean values, for birth length (Figure 7B), exponential growth rate (Figure 7D), and asymmetry between daughters (Figure 7E). Interestingly, variation in birth length for old-pole daughters grown in glycerol is less (14.93–18.13%) than variation in new-pole daughters (17.91–20.67%), which is surprising given the enhanced asymmetry for old-pole cells (Figure 7E), as well as the dependence of birth length on pole age (Figure 4C). This may be explained by the observation that asymmetry is only derived from inheritance of the old-pole, regardless of its age (Figure S8), thus while pole age increases the birth size of old-pole inheritors, it does not affect the asymmetry between new- and old-poles. As a consequence, new-pole daughter birth length must increase with the pole age of their old-pole sibling which may lead to increased observed variability in new-pole daughters compared to old-pole daughters in the population. This effect is not observed in the birth lengths of cells grown in acetate or pyruvate (Figure 7B), with similar CVs, which may reflect both reduced asymmetry (Figure 7E) and sizer-like behavior (Figure 7A).

It is also notable that variation in birth length for cells grown in acetate (Figure 7B) is not significantly different between new- and old-pole inheritors, given the different relationship between division length and birth length (Figures 5C,D, 7A) since a sizer-based cell size control mechanism implies that there should be reduced noise in birth lengths. This expected variation in noise given the disparity between new-pole sizers and old-pole adders may however be compensated by the reduced asymmetry in division observed for cells grown in acetate (Figure 7E). The differences in asymmetry between glycerol, acetate, and pyruvate may further explain the lack of scalability observed in birth length (Figure S7B). We also note that the noise in division even after accounting for asymmetry between new- and old-pole cells is still much higher that what is observed in *E. coli* particularly for growth in glycerol.

### 3.6. Effects of inheritance on cell size control

**Figure 8:**
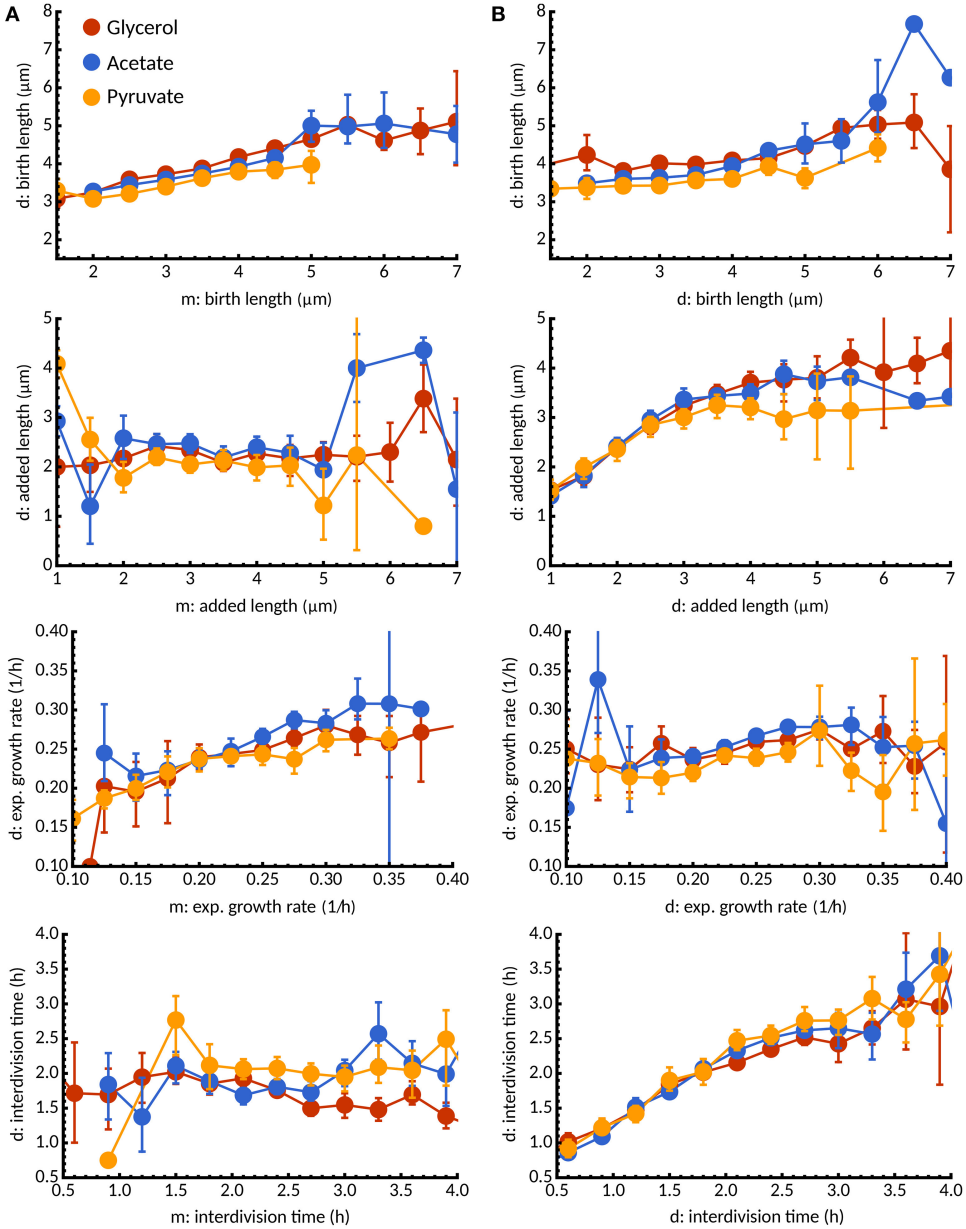
Inheritance of growth parameters under different carbon sources. **(A)** Conditional expectation of birth length, added length, exponential growth rate, and interdivision time for daughter cells (d) as a function of the corresponding growth parameter in the mother cell (m). Colors indicate different carbon sources: glycerol (red), acetate (blue), pyruvate (yellow). For all media, we observe a statistical dependence between birth lengths in mother and daughter cells consistent with the adder principle but no inheritance between added lengths. Interestingly, growth rate is inherited but not interdivision time. **(B)** In contrast, between sister cells added length and interdivision times are statistically dependent but not growth rate and interdivision times.

Given that cell size control robustly obeys the adder principle at the population level for at least two carbon sources, we sought to investigate the constraints of heritability on cell size control. In perfect adders, for example, maintaining cell size homoeostasis requires the added length of daughter cells to be independent of added length of mothers. Indeed, we find there is no significant dependence for any of the three conditions, but birth lengths in daughters show some positive correlation with the corresponding lengths of mothers (Figure 8A). Both of these observations for all three carbon sources are consistent with cells obeying the adder principle although—reflecting more sizer-like properties in pyruvate—this relationship is less strong compared to acetate and glycerol cells. Interestingly, we found that growth rates of daughters correlate positively with the mother growth rate but there is no significant correlation between their interdivision times. These relationships between mother and daughter cells were unchanged when new- and old-pole inheriting cells were analyzed separately (data not shown).

Despite the effects of mother-daughter correlations, decisions made in the mother cell could potentially affect both daughter cells in similar ways. To investigate this issue, we studied the statistical dependence between sister cells. Surprisingly, we found that added lengths and interdivision times were highly similar between sister cells, in contrast to birth lengths and growth rates (Figure 8B). These observations highlight that while growth rates are mostly inherited, interdivision times and added length are already set in the mother cell. Multiple rounds of DNA-replications (as observed in Santi and McKinney, 2015) could potentially account for such effects but are beyond the scope of our study. We found that the above considerations are unchanged when new- and old-pole subpopulations are considered separately under all three conditions (data not shown).

## 4. Discussion

Microfluidics coupled with time-lapse microscopy is a powerful tool for determining the relationships between size and growth in single cells. Here, we have studied mycobacteria growing on three different carbon sources and showed that they adapt their cell-size regulation depending on the growth conditions. Various measures of single-cell growth have been used in the literature, including linear (e.g., Aldridge et al., 2012) and exponential models (e.g., Wakamoto et al., 2013). The method by which growth rate is assessed can lead to significantly different conclusions based on the same data, thus we have determined this quantitatively using statistical model selection. This analysis reveals that in approximately 90% of cells, independent of carbon source, single-cell growth is more accurately described as exponential growth rather than linear growth (Figure 1). This strongly implies that single-cell growth in *M. smegmatis* is exponential, in agreement with other authors who have shown that single-cell elongation is size-dependent (Santi et al., 2013). Using this information, we are able to interpolate between images in our timelapse data and correct inaccuracies introduced by the sampling frequency (images are acquired every 15 min). Thus—by using fitted exponential growth rates—birth lengths, division lengths, and interdivision times can be estimated, resulting in more accurate overall estimates of single-cell parameters.

We show here, by determining the slope of the linear regression line of division length against birth length, that *M. smegmatis* cells follow the adder principle when grown with glycerol as a sole carbon source (Figure 2A). For 544 observed birth and division events, the slope is 0.996 ± 0.102 which is consistent with an adder model, indicating that on average cells add a constant length between birth and division (3.525 ± 0.453 μm). This is in agreement with existing reports from other authors who have demonstrated that *M. smegmatis* cells are not sizers (Aldridge et al., 2012), or are neither sizers nor timers (Santi et al., 2013). We note that these other authors have used similar experimental approaches to this report, using microfluidic devices coupled with time-lapse microscopy (Aldridge et al., 2012; Santi et al., 2013), though using 7H9 medium rather than HdB as here. We observe very similar growth rates and size distributions and are confident that our conditions are sufficiently similar to allow detailed comparison (Figure 2; Aldridge et al., 2012; Santi et al., 2013). Interestingly, the above authors had some discrepancies when determining the dependence of added length on birth length, with Aldridge et al. (2012) reporting no relationship and Santi et al. (2013) reporting a minor dependence (*r* = 0.20). Here, we observe no relationship between birth length and added length, with *r* = −0.003 in agreement with Aldridge et al. (2012) though the same authors also report that *M. smegmatis* microcolonies appear to synchronise their cell divisions, with significant differences in mean interdivision times between individual microcolonies. Under our conditions we do not observe significant differences between microcolonies with *p* = 0.520 (Kruskal-Wallis H-test), suggesting that interdivision time is a universal parameter related to the population growth rate rather than a single cell variable (Figure S2). Taken together, these data therefore imply that *M. smegmatis* cells are adders in the strictest sense, in that added length is independent from birth length, when grown in glycerol as a sole carbon source.

Given that *M. smegmatis* cells are adders when grown in glycerol, we observe interesting dependencies between single-cell parameters. Added length in *M. smegmatis* cells appears to depend on exponential growth rate (Figure 2C) with faster growing cells adding more length prior to division. We found that a similar dependence holds for *E. coli* based on the dataset of Taheri-Araghi et al. (2015) (data not shown; personal communication with Sattar Taheri-Araghi). This is coupled with the observation that growth rate is dependent on birth length (Figure 2D), with smaller cells growing faster than larger cells at birth (for cells grown in glycerol but not for cells grown in acetate or pyruvate; Figures 5B, 6B); a dependence that is not observed in *E. coli*. How these correlations affect the properties of cell size control is not clear and should be the subject of further modeling studies (Modi et al., 2016), though we note that the striking negative dependence of growth rate on birth length has recently been observed in mammalian cells (Ginzberg et al., 2017).

Mycobacteria extend exclusively from their cell poles (Thanky et al., 2007; Joyce et al., 2012), and mounting evidence points to preferential incorporation at one pole, with the inheritance of this growth pole (the “old-pole”) leading to asymmetry with increased cell length, enhanced linear elongation rate, and potentially variable antibiotic susceptibility (Aldridge et al., 2012; Santi et al., 2013; Wakamoto et al., 2013; Manina et al., 2015; Richardson et al., 2016). We also observe significant differences in birth and division length between daughters inheriting the new- or old-pole, as well as differences in linear elongation rate (Figure 3). We note that in contrast with Wakamoto et al. (2013) and Santi et al. (2013), we observe minor but significant differences in exponential growth rate between pole inheritors, with new-pole inheritors having a marginally higher exponential growth rate (0.271 ± 0.110 h^−1^) that old-pole inheritors (0.257 ± 0.800 h^−1^; Figure 3), a result that is consistent with recent data for *M. tuberculosis* single cells (Manina et al., 2015).

Given these differences in size and growth, we sought to determine whether these factors had an effect on cell size control in *M. smegmatis* single cells. By separating *M. smegmatis* cells at division based on whether they inherit the old-pole or new-pole of their mother cell, we can determine relationships between division length and birth length as performed for the whole dataset. We find that the slope of the linear regression line is not significantly different between the two sub-populations (0.948 ± 0.162 and 0.894 ± 0.149) for old- and new-pole inheritors respectively; Figures 4A,B). Moreover, when the old-pole sub-population is further sub-divided by absolute pole age, there is no significant deviation from the adder principle as pole age increases (Figure 4E). These results indicate that when grown with glycerol as a sole carbon source *M. smegmatis* single cells behave as adders irrespective of pole age, despite significant differences in cell size and growth.

Previous investigations of cell size control in mycobacteria have exclusively used standard laboratory medium, whether 7H9 or HdB, supplying glycerol (Aldridge et al., 2012) or glucose and glycerol (Santi et al., 2013; Wakamoto et al., 2013; Manina et al., 2015) as carbon sources. These sources are thought to result in optimal growth rates for *M. smegmatis* cells grown *in vitro*, but are unlikely to reflect physiological sources of carbon for mycobacteria. We thus sought to determine how less optimal carbon sources might affect the growth and cell size control of *M. smegmatis* cells. Recent evidence suggests that *M. tuberculosis* cells survive intracellularly within macrophages by catabolizing lipids such as cholesterol (Salamon et al., 2014; Vromman, 2014), of which the metabolic products are pyruvate and acetyl-CoA (via propionyl-CoA). There is significant evidence that both virulence and dormancy programmes in *M. tuberculosis* are dramatically affected by the available carbon source, for example with mycobacterial surface lipids being remodeled (Jain et al., 2007). Here, we demonstrate that carbon source can affect cell size control, and thus must be taken into consideration for future investigations into mycobacterial physiology.

We use HdB supplemented with pyruvate or acetate as sole carbon sources in order to determine the impact of such changes on cell size control. At the population level, we clearly observe an increase in doubling time when measured by either optical density or colony-forming-units, although differences in optical density between pyruvate and acetate cultures are not reflected by CFU counts (Figure S6). These inconsistencies not only imply that alternative carbon sources reduce the mean population growth rate, but also that mean cell size is altered between pyruvate and acetate cultures. When cells are observed in the microfluidic device, there is a clear increase in interdivision time for cells grown in acetate or pyruvate compared to cells grown in glycerol, as well as a decreased exponential growth rate for cells grown in pyruvate (Table 1). We determined how well cells grown in these alternative carbon sources conformed to the adder principle as seen when grown in glycerol, and observed marked changes in the relationship between division and birth length (Figure 7A).

When grown in acetate, the slope of regression line between birth and division length is 0.964 ± 0.140 (*n* = 383; Figure 5A), which is fully consistent with the adder principle. However, when separating cells by the age of the pole inherited, significant differences emerge, with a slope of 1.181 ± 0.212 (*n* = 197) for old-pole inheritors, but a slope of 0.682 ± 0.187 (*n* = 186) for new-pole inheritors which is not consistent with a perfect adder principle (Figures 5C,D). These values imply that old-pole inheritors, while consistent with adder, have some timer-like characteristics, and that new-pole inheritors are not consistent with adders, with sizer-like properties. This is especially noticeable given that asymmetry between new- and old-pole inheritors is reduced compared to glycerol (Figures 7B,E), which might suggest that there would be fewer differences in cell size control. A key prediction of the sizer principle, where cells divide close to some threshold length is that fluctuations around this threshold are rapidly corrected in the next generation, implying that noise in birth length should be reduced in a sizer cell compared to a pure adder. However, while we observe a reduction in noise for all cells, we do not observe differences in the coefficient of variation of birth length for new-pole compared to old-pole cells (Figure 7B), though this may be masked by the reduction in overall asymmetry compared to cells grown in glycerol.

Cells grown with pyruvate as a sole carbon source display a significant deviation from perfect adders regardless of which pole is inherited, which may reflect more pronounced changes in single-cell growth parameters: cells have reduced linear elongation rate, reduced exponential growth rate, and increased interdivision time compared to cells grown in acetate or glycerol (Table 1). As a population, the slope of birth length against division length is 0.832 ± 0.137, a deviation from the adder principle which is retained in new- and old-pole cells with slopes of 0.815 ± 0.221 and 0.767 ± 0.192 respectively (Figure 6). Interestingly, a recent paper on cell size control in *E. coli* has observed that cell size control appears sizer-like at slow growth and adder-like at fast growth (Wallden et al., 2016) which appears to be in agreement with these data, although their differences in growth rate were far greater compared to this study. As with acetate, noise in birth length is reduced compared to glycerol, and consistent with sizer-like properties, especially given that asymmetry is similar in pyruvate compared to glycerol (Figures 7B,E).

There has been renewed interest in the age-structure observed in microfluidic experiments with significant variations in cell division rates (Powell, 1956; Hashimoto et al., 2016; Thomas, 2017). In cell populations, such fluctuations cause over-representation of cells that divide at a faster rate than other cells. We show that lineage-weighting of tree structured data can be used to reduce this bias. We observe differences between population and lineage-weighted distributions in all carbon sources, with reduced interdivision time in the population statistics compared to lineages, as expected (Figure S3F). However, differences in exponential growth rate between population and lineage statistics vary between carbon sources. An intuitive expectation of reduced interdivision time in the population statistics is that exponential growth rate should concurrently increase—cells which divide faster should grow faster. However, we only observe this effect in cells grown in acetate and pyruvate, but not glycerol (Figure S3E). This may be explained by the dependence of interdivision time on exponential growth rate, which shows no dependence for glycerol, but a negative dependence in both acetate and pyruvate (Figure S4F2). This demonstrates that in glycerol, fast-growing cells do not divide more frequently than slow-growing cells, but do so in acetate and pyruvate. This observation is surprising, given that for cells dividing after adding a constant length, the factor limiting division timing is growth rate. Yet we observe that although added length is independent of birth length, it varies with the exponential growth rate.

The molecular mechanism underlying cell size control remains an open question, but several authors have proposed a model by which a protein accumulates proportionally to cell size, and triggers division after reaching a threshold (Fantes et al., 1975; Ghusinga et al., 2016). Such a model can explain both adder and sizer principles, where degradation of the protein at cell birth leads to adder, and equal sharing leads to sizer (Deforet et al., 2015; Bertaux et al., 2016). There is also extensive debate regarding the precise point at which the adder principle is applied, with suggestions that it applies solely from birth to division (e.g., Campos et al., 2014), or recent evidence that it applies at the initiation of replication (Ho and Amir, 2015; Taheri-Araghi et al., 2015; Si et al., 2017). However, we are unable to speculate on the applicability of this concept in mycobacteria since we have not tracked DNA replication.

Here we have used detailed analysis of microfluidic single-cell experiments to show that cell size control in mycobacteria varies with carbon source, and that inherent asymmetry in division causes effects that are not observed in existing model species. Previous investigations of cell size control in mycobacteria have exclusively used standard laboratory medium, utilizing carbon sources that are unlikely to be available naturally. We demonstrate that utilizing glycerol, mycobacteria behave as adders, but when provided with alternative carbon sources, can modify this behavior. The inherent heterogeneity introduced by asymmetric division also appears to cause deviations from the general principles observed in *E. coli*, but also display characteristics not observed in other asymmetric species such as *C. crescentus*. We believe that further investigation of the relationship between single-cell growth and population heterogeneity may inform reports of variable antibiotic tolerance in mycobacteria.

## Author contributions

MP performed the experiments; MP and PT analyzed the data; MP, PT, BR, and VS designed the experiments and wrote the manuscript.

### Conflict of interest statement

The authors declare that the research was conducted in the absence of any commercial or financial relationships that could be construed as a potential conflict of interest.
