# Supplementary material for: Mycobacteria Modify Their Cell Size Control under Sub-Optimal Carbon Sources
Source: Front Cell Dev Biol. 2017 Jul 12;5:64. doi: 10.3389/fcell.2017.00064 (PMC5506092; doi:10.3389/fcell.2017.00064)
Supplement: Supplementary file 2 [file DataSheet1.PDF]

Supplementary Material: Mycobacteria modify their cell size control under sub-optimal carbon sources.

Miles Priestman [1], **Philipp Thomas** [2], **Brian D. Robertson** [1,*], **Vahid Shahrezaei** [2,*]

*Correspondence:
Vahid Shahrezaei
v.shahrezaei@imperial.ac.uk

Brian D. Robertson
b.robertson@imperial.ac.uk

SUPPLEMENTARY TABLES

Table S1. Mean and standard deviations for all growth parameters in glycerol, acetate, and pyruvate. Attached as separate spreadsheet.

SUPPLEMENTARY FIGURES

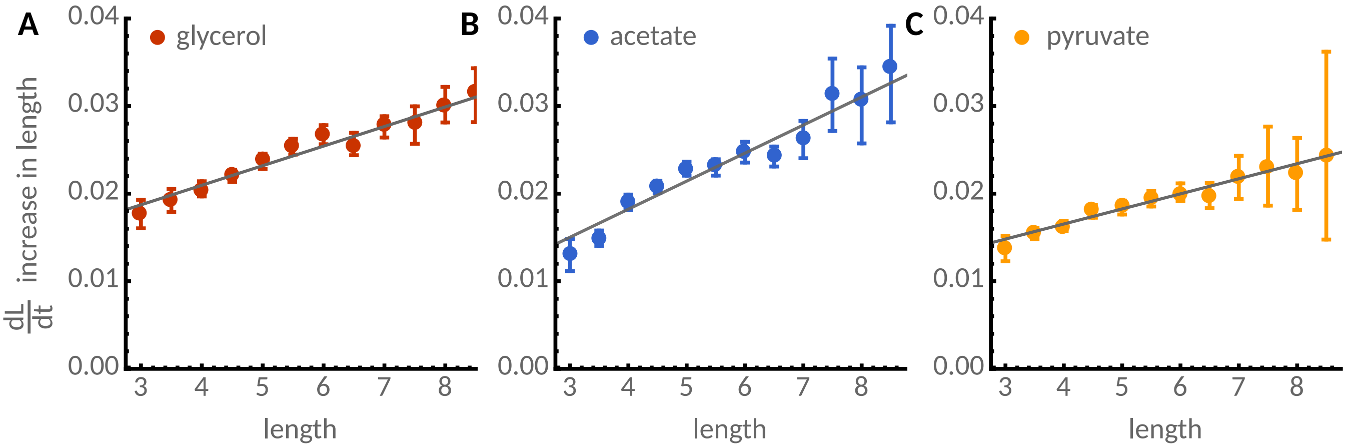

Figure S1. *M. smegmatis* cells elongate in a length-dependent manner. Average increase of cell length, measured by finite-difference derivative, scales linearly with cell length in glycerol (A), acetate (B) and pyruvate (C), which sugguests exponential growth.

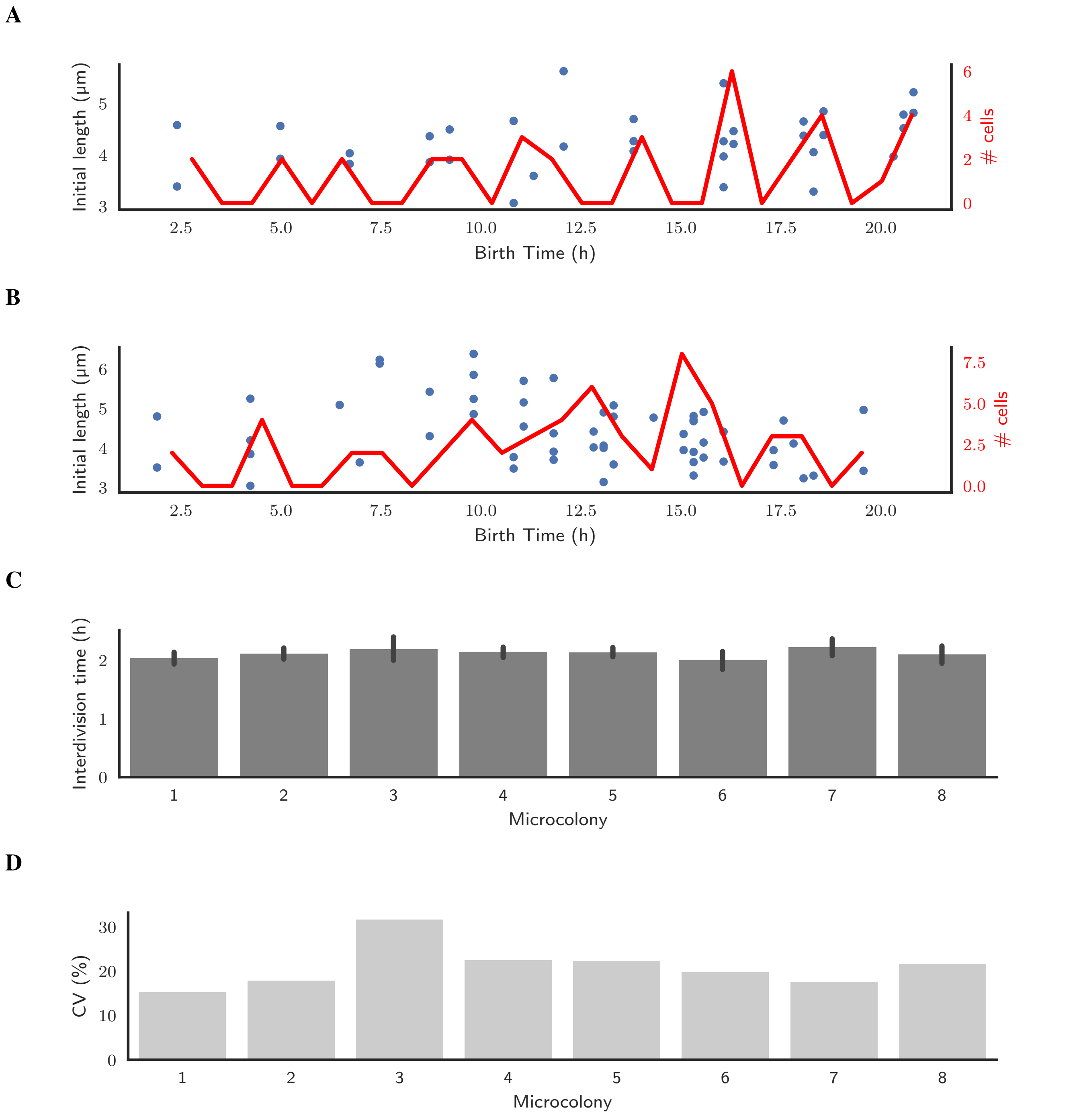

Figure S2. Analysis of interdivision time synchronisation within single-cell microcolonies. (A,B) Depiction of two example microcolonies founded by a single cell with birth length plotted against time of birth over the course of an experiment. Blue circles represent individual division events observed; the red line represents a frequency histogram of cell numbers with a bin size of 0.75 h. (C) For each of 8 individual microcolonies, the mean interdivision time of all cells within each lineage is shown $\pm$ the 95 % confidence interval. n-values are 35, 56, 44, 110, 125, 24, 24, and 35, in ascending order of microcolony number. (D) Coefficient of variation of interdivision time across each microcolony (CV = % s.d. / mean). n-values as above.

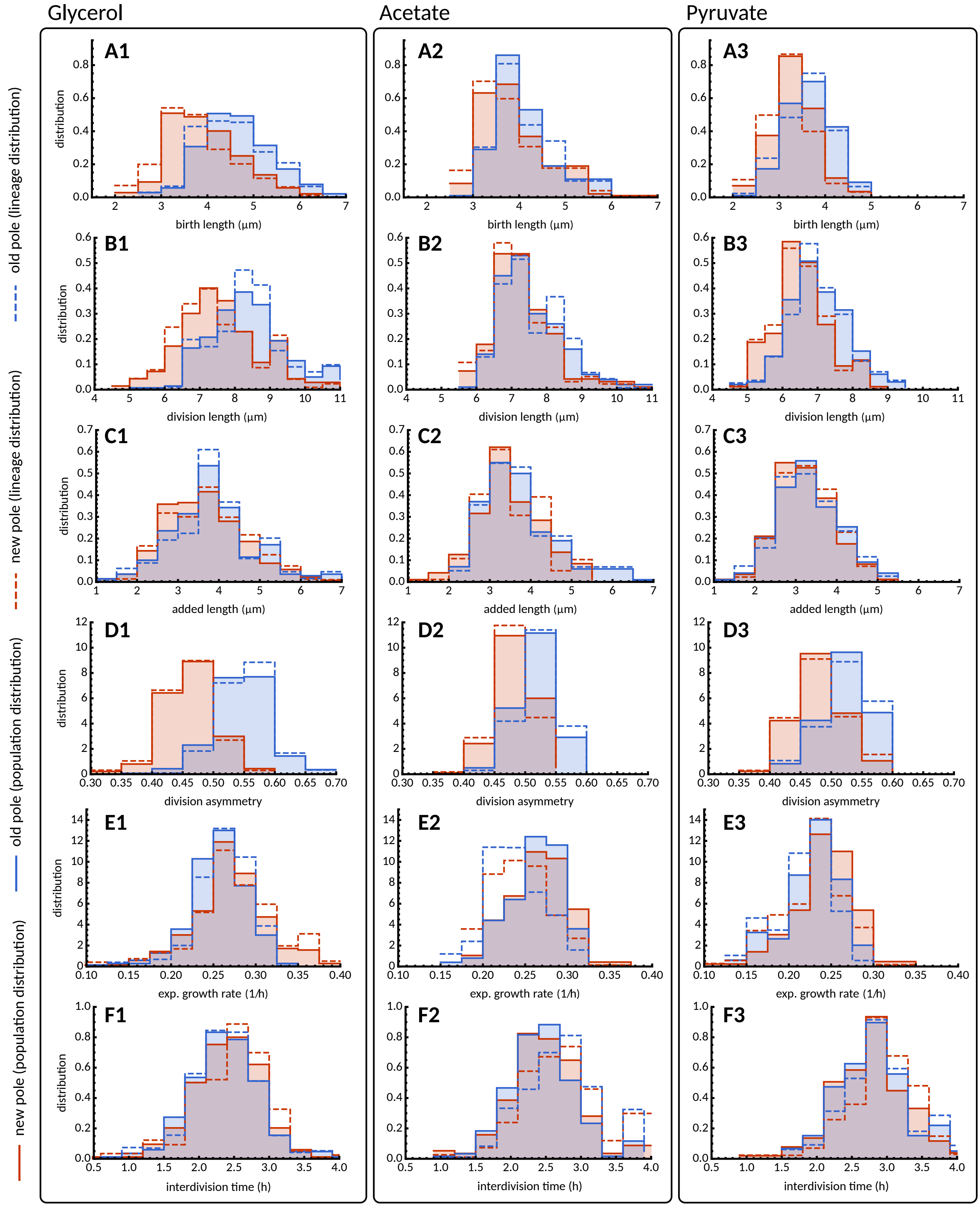

Figure S3. Distributions of growth parameters for cells grown with different carbon sources. Histograms of birth length, division length, added length, division asymmetry, exponential growth rate and division rate are shown for new (red) and old pole cells (blue) grown in glycerol (A1-F1), acetate (A2-F2) and pyruvate (A3-F3). Population average distributions taken over every single cell in the experiment (solid lines) are compared to lineage-weighted distributions (dashed lines). Cells in the population are biased towards shorter interdivision times compared to the lineage-weighted statistics (F1-F3). Similarly, the exponential growth rates are higher in the population than in lineages (E2 and E3) except for glycerol (E1).

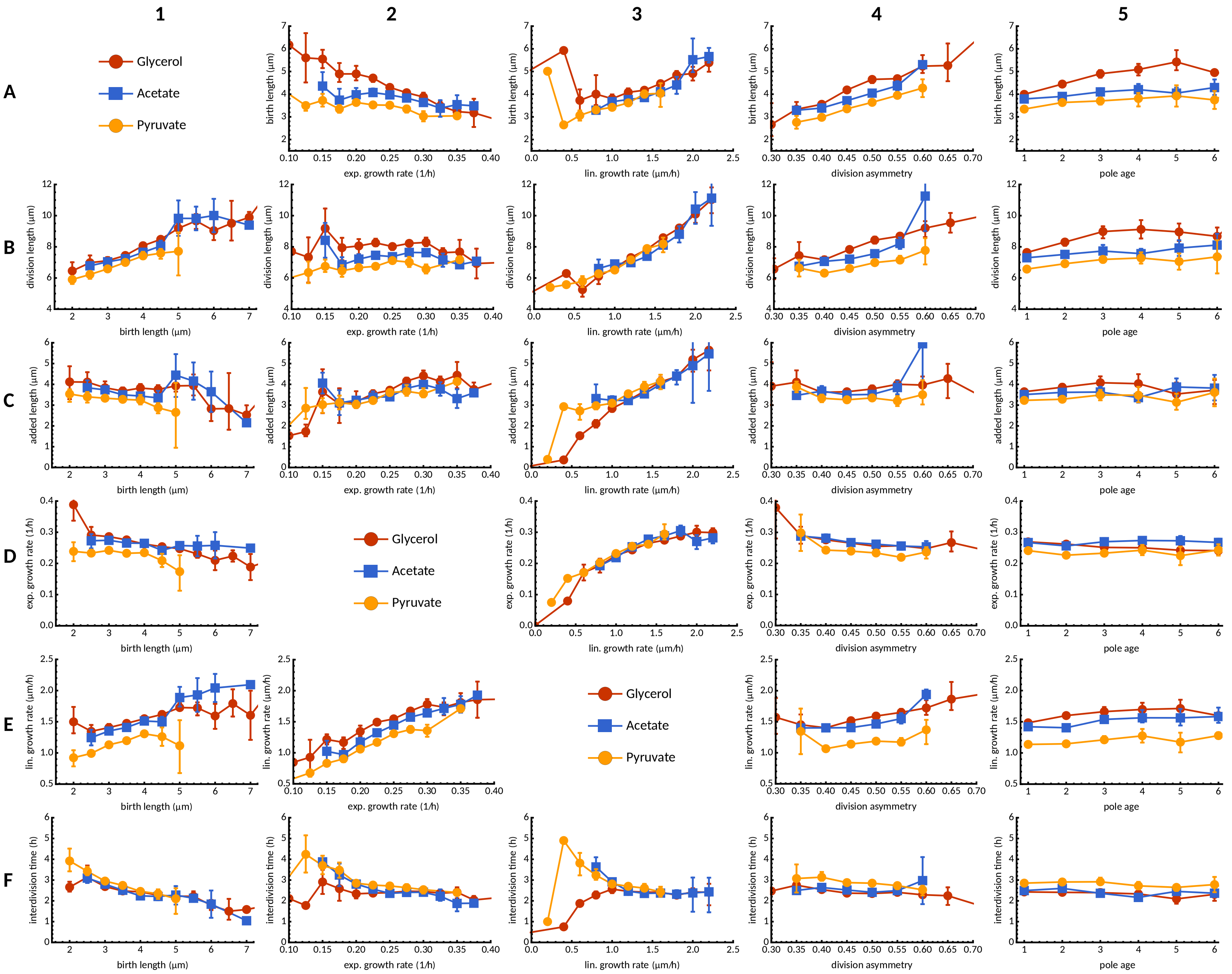

Figure S4. Interdependence of growth parameters for cells grown with different carbon sources. Mean of growth parameters binned according to birth length (column 1), exponential (2) and linear growth rate (3), division asymmetry (4) and pole age (5) for cells grown in glycerol (red), acetate (blue) and pyruvate (yellow). Error bars denote 95% bootstrap-confidence intervals.

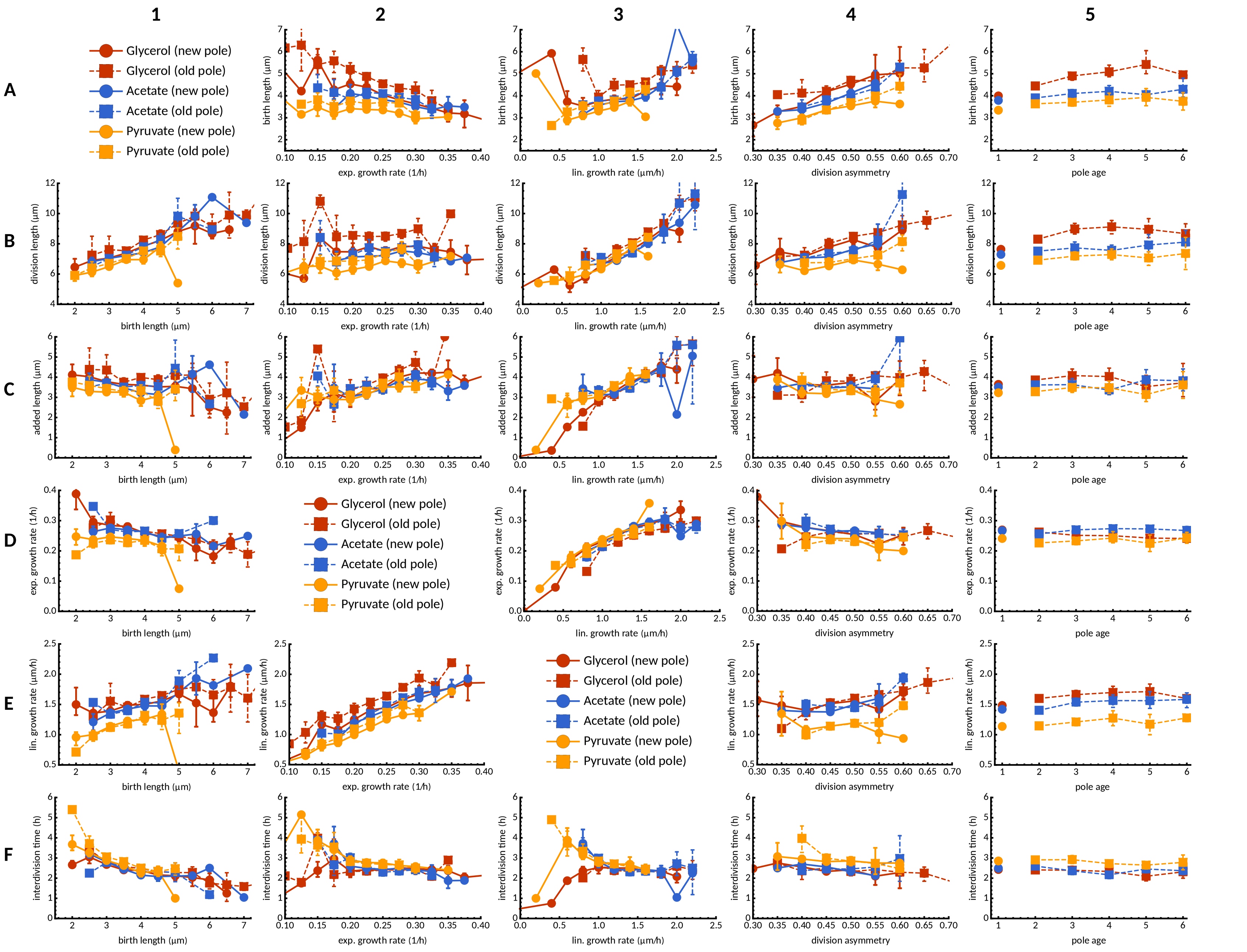

Figure S5. Interdependence of growth parameters for cells sorted by pole age. Same as in Figure S4 but distinguishing old- (circles) and new pole cells (squares).

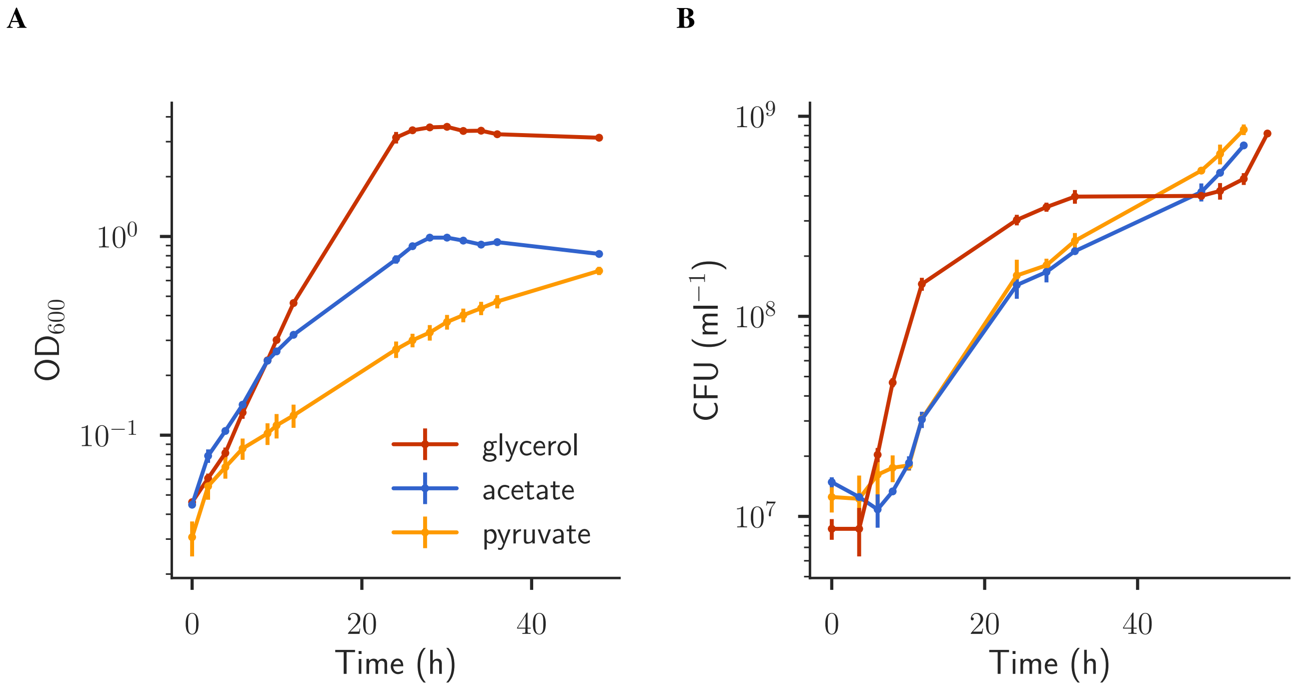

Figure S6. Growth curves for *M. smegmatis* cells grown in various carbon sources. (A) As measured by optical density at 600 nm. Doubling times as estimated from the exponential phase of the growth curves were 3.4 h, 4.8 h, and 8.6 h for glycerol, acetate, and pyruvate respectively. (B) As measured by colony-forming units. Doubling times as estimated from the exponential phase of the growth curves were 2.0 h, 4.9 h and 4.7 h for glycerol, acetate, and pyruvate respectively.

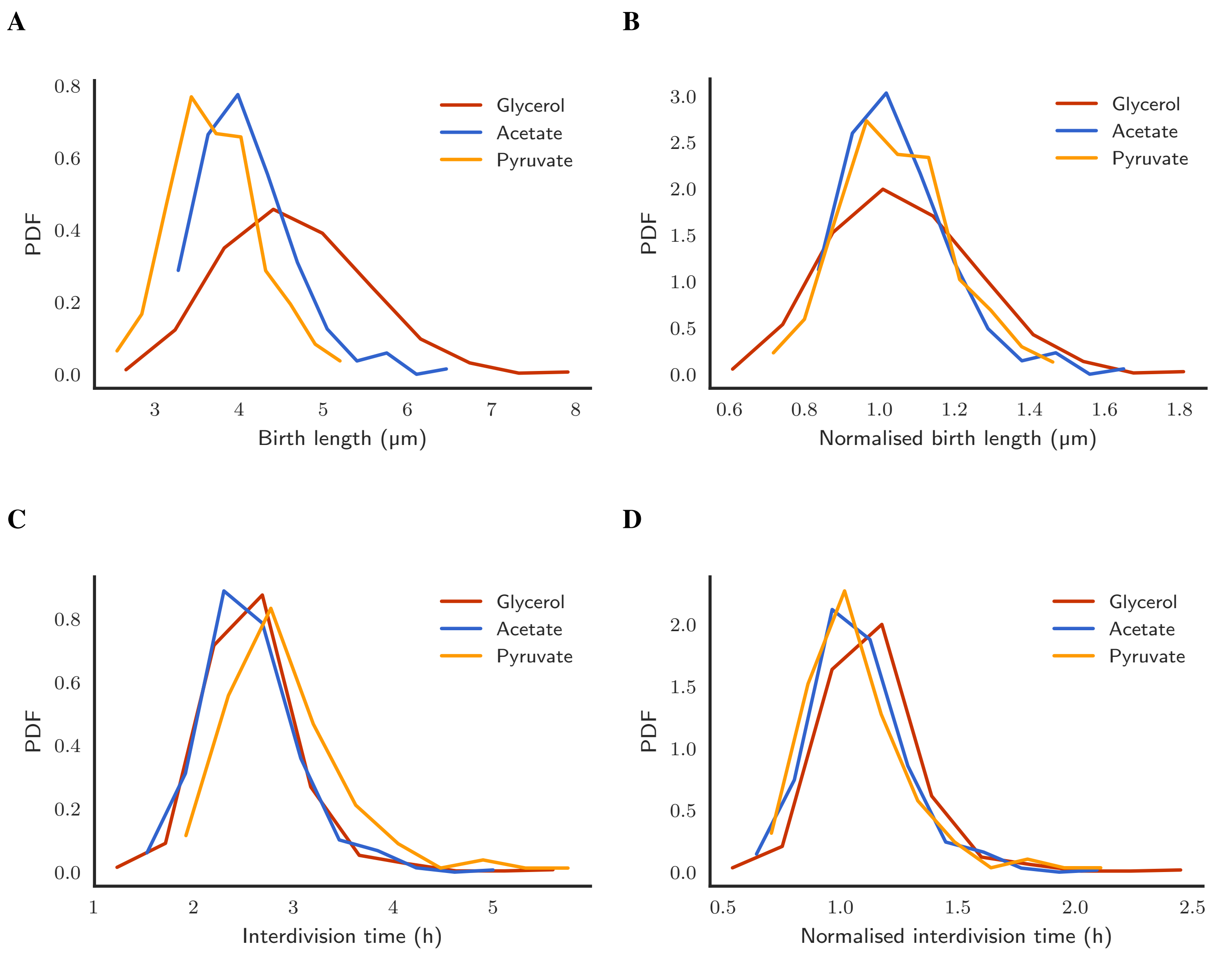

Figure S7. Scaling of birth length and interdivision time distributions for *M. smegmatis* cells grown in different carbon sources. Plots depict histograms for single cells grown in glycerol, acetate, or pyruvate as a sole carbon source with different means. (A) Distribution of birth length for all cells. (B) Birth length of all cells scaled by mean birth length. (C,D) Interdivision time of all cells unscaled and scaled by mean respectively.

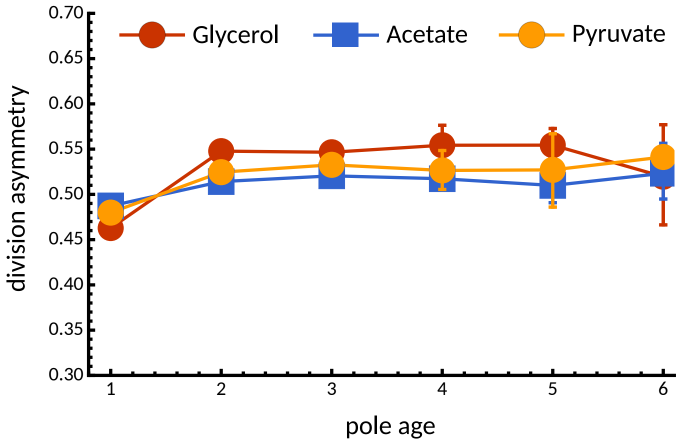

Figure S8. Division asymmetry derives from inheriting the old pole regardless of its age. The average fraction of length inherited after cell division (division asymmetry) is shown. New pole cells (age 1) inherit less length than old pole cells on average. Asymmetry is practically constant for old poles of various ages (> 1).
